# 5-Oxo-dihydropyranopyran derivatives as anti-proliferative agents; synthesis, biological evaluation, molecular docking, MD simulation, DFT, and *in-silico* pharmacokinetic studies

**DOI:** 10.1016/j.heliyon.2024.e29850

**Published:** 2024-04-23

**Authors:** Sara Ranjbar, Paria Sadeghian, Sara Khademian, Mina Emami, Zahra Pakrouh Jahromi, Seyedeh Habibeh Mirmajidi, Fateme Zare, Manica Negahdaripour, Younes Ghasemi, Mehdi Khoshneviszadeh

**Affiliations:** a Pharmaceutical Sciences Research Center, Shiraz University of Medical Sciences, Shiraz, Iran; b Computational Vaccine and Drug Design Research Center, Shiraz University of Medical Sciences, Shiraz, Iran; c Department of Medicinal Chemistry, School of Pharmacy, Shiraz University of Medical Sciences, Shiraz, Iran; d Department of Medical Biotechnology, School of Advanced Medical Sciences and Technologies, Shiraz University of Medical Sciences, Shiraz, Iran; e Department of Pharmaceutical Biotechnology, School of Pharmacy, Shiraz University of Medical Sciences, Shiraz, Iran

**Keywords:** Dihydropyrano[4,3-*b*]pyran, Cytotoxicity, Anti-cancer, Cyclin-dependent kinase, Drug-likeness, Molecular dynamics simulation, CDK2

## Abstract

A series of ethyl 2-amino-7-methyl-5-oxo-4-phenyl-4,5-dihydropyrano[4,3-*b*]pyran-3-carboxylate derivatives (**4a**-**j**) bearing different substitutions on the C_4_-phenyl ring was synthesized. The anti-proliferative activity of all the synthesized compounds was assessed against two human cancer-cell lines, including SW-480 and MCF-7, by using MTT method. Derivatives **4g**, **4i**, and **4j**, possessing 4-NO_2_, 4-Cl, and 3,4,5-(OCH_3_)_3_ substitutions, were found to be the most potent compounds against both cell lines. The obtained IC_50_ values for **4g**, **4i**, and **4j** were 34.6, 35.9, and 38.6 μM against SW-480 cells and 42.6, 34.2, and 26.6 μM against MCF-7 cells, respectively. Evaluation of the free radical scavenging potential of the compounds against DPPH radicals showed the highest result for compound **4j** with an EC_50_ value of 580 μM. Molecular docking studies revealed the compounds were well accommodated within the binding site of cyclin-dependent kinase-2 (CDK2) with binding energies comparable to those of DTQ (the co-crystallized inhibitor) and BMS-265246 (a well-known CDK2 inhibitor). Molecular dynamics simulation studies confirmed the interactions and stability of the **4g**-CDK2 complex. All derivatives, except **4g**, were predicted to comply with the drug-likeness rules. Compound **4j** may be proposed as an anti-cancer lead candidate for further studies due to the promising findings from *in-silico* pharmacokinetic studies, such as high GI absorption, not being a P-gp substrate, and being a P-gp inhibitor. Density functional theory (DFT) analysis was performed at the B3LYP/6–311++G (d,p) level of theory to examine the reactivity or stability descriptors of **4d**, **4g**, **4i**, and **4j** derivatives. The highest value of energy gap between HOMO and LUMO and thermochemical parameters were obtained for **4i** and **4j**.

## 1 Introduction

Since the start of the twenty-first century, cancer has become a major leading cause of death globally [1]. The incidence and mortality of cancer increases every year. The global cancer statistics indicate 19.3 million newly diagnosed cases and 10 million cancer-caused deaths worldwide in 2020 [2]. Cancer prevalence is predicted to increase rapidly reaching 13.1 million cases in 2030 [3]. The most significant attributes of cancer are uncontrolled proliferation, local invasion, and distant metastasis. Most cancer-related deaths are caused by tumor recurrence or distant metastasis after systemic antitumor therapy [4]. Traditional treatments such as surgery, chemotherapy, radiotherapy, and immunotherapy are associated with several side effects that can lead to systemic adverse effects [5]. The main disadvantages of systemic chemotherapy include low drug concentration in the tumor, rapid clearance from the circulation, and severe toxicity outside the tumor [6]. Furthermore, some anti-cancer drugs are prone to resistance or have a short half-life due to rapid degradation by enzymes [6,7]. Considering these limitations, scientists still endeavor to develop more effective small chemotherapeutic molecules with fewer side effects.

**Fig. 1:**
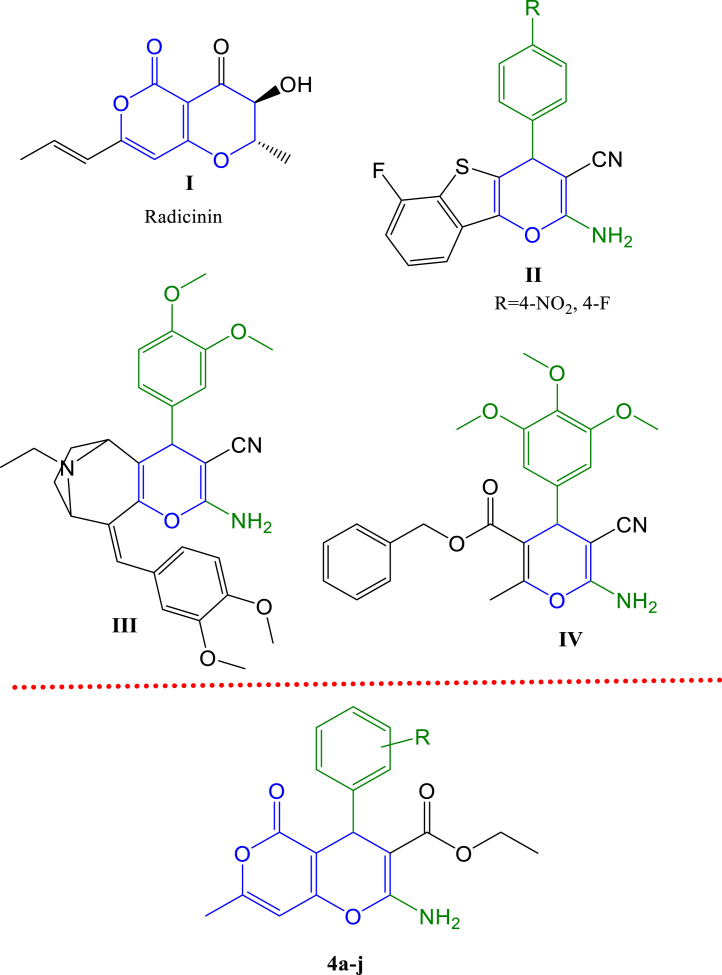
Examples of pyran-containing natural (**I**) and synthetic compounds (**II-IV**) with anti-cancer activities and the general structure of the target compounds of this study (**4a-j**).

The pyran ring is a core structural unit in many natural and synthetic compounds [8,9]. Pyran derivatives have shown various biological activities such as antibacterial, antifungal, antitubercular, antimalarial, anti-inflammatory, antidiabetic, antityrosinase, and antioxidant properties [[10], [11], [12], [13], [14], [15], [16]]. Moreover, the pyran core has emerged as a promising pharmacophore due to the anti-cancer capabilities of pyran-based natural and synthetic derivatives [8,17,18]. Radicinin (Fig. 1, **I**), a dihydropyranopyran-4,5-dione produced by *Cochliobolus australiensis*, exhibited a promising anti-cancer activity with IC_50_ values of 60.68, 30.89, and 87.89 μg/mL against WiDr, T47D, and HeLa cells, respectively [19,20]. Previous research revealed that cyclin-dependent kinase-2 (CDK2) is a valid anti-cancer target for pyran-containing compounds [[21], [22], [23], [24]]. As shown in Fig. 1, 4*H*-pyran derivatives **II** and **III** exhibited antitumor and CDK2 inhibitory activities [22,23]. Moreover, a dihydropyranopyran derivative (Fig. 1, **IV**) was reported as a potential CDK2 inhibitor showing cytotoxic activity against HCT-116 cells with an IC_50_ value of 75.10 μM [24].

This study aimed to synthesize a series of substituted dihydropyranopyran derivatives (Fig. 1, **4a-j**) as anti-cancer agents and investigate their cytotoxicity against two cancer cell lines. Molecular docking analysis is performed to evaluate the binding energies, modes, and compounds interactions within the active site of CDK2. Besides, the drug-likeness and pharmacokinetic properties of the derivatives are predicted *in-silico*. Finally, DFT analysis is carried out to determine the reactivity or stability as well as the nucleophilic and electrophilic sites of the studied compounds [25], using the B3LYP method with the 6–311++G (d,p) basis set.

## 2 Materials and methods

### 2.1 Chemicals and apparatuses

The chemicals were purchased from Sigma-Aldrich (St. Louis, MO) and used without further purification. Reactions were monitored by thin layer chromatography (TLC) on MERCK precoated silica gel 60-F254 (0.5 mm) aluminum plates. Melting points were measured on a Kofler hot stage apparatus and were uncorrected. The Nuclear magnetic resonance (NMR) spectra were recorded on a Bruker 300 MHz NMR spectrometer with tetramethylsilane (TMS) as an internal standard. ^1^H NMR (300 MHz) and ^13^C NMR (75 MHz) spectra were recorded in DMSO‑*d*_6_. Chemical shifts (*δ*) were expressed in parts per million (ppm) unit, and *J*-coupling values were reported in Hertz (Hz). Mass spectra were determined with an Agilent spectrometer (Agilent Technologies 9575c inert MSD, USA). Infrared spectra (IR) were recorded on an FT-IR PerkinElmer Precisely system spectrophotometer (PerkinElmer, Waltham, MA) using the KBr disc technique.

### 2.2 Synthesis of ethyl 2-amino-7-methyl-5-oxo-4,5-dihydropyrano[4,3-b]pyran-3-carboxylate derivatives (**4a-j**)

4-Hydroxy-6-methyl-2-pyrone (1 mmol; 126 mg), ammonium acetate (30.5 mmol; 8.5 mg), ethyl cyanoacetate (1 mmol; 110 μL), and corresponding benzaldehyde (1 mmol) were poured into a flask containing water and ethanol (8 mL; 1:1) and refluxed for 24 h. The progress and completion of the reaction were checked through TLC by using chloroform-ethanol with a ratio of 9 %–1 % as the mobile phase. Then, the flask was placed in an ice container. The formed precipitate was washed several times with water and petroleum ether to yield the pure product. In the case of compound **4j**, the pure product was obtained after TLC by using chloroform-ethanol (90:10) as the eluent.

#### 2.2.1 Ethyl 2-amino-7-methyl-5-oxo-4-phenyl-4,5-dihydropyrano[4,3-b]pyran-3-carboxylate (**4a**)

The compound was obtained as cream crystals with a yield of 47.6 % and had a melting point range of 145–148 °C; *Rf* = 0.46 (EtOH/CHCl_3_ 1:9, v/v); ^1^H NMR (DMSO‑*d*_6_, 300 MHz) *δ*_H_ (ppm): 1.07 (t, 3H, *J* = 4.2 Hz, CH_2_–CH_3_), 2.21 (s, 3H, C_7_–CH_3_), 3.95 (q, 2H, *J* = 4.2 Hz, CH_2_–CH_3_), 4.54 (s, 1H, C_4_–H), 6.28 (s, 1H, C_8_–H), 7.12–7.25 (m, 5H, phenyl-H_2-6_), 7.71 (s, 2H, NH_2_); ^13^C NMR (DMSO‑*d*_6_, 125 MHz) *δ*_C_ (ppm): 14.44, 19.74, 34.92, 59.42, 77.58, 98.39, 104.06, 126.68, 129.76, 131.24, 145.83, 158.31, 159.19, 162.25, 162.90, 168.16; (EI) *m/z* (%): 327 (M^+^, 20), 255 (16), 250 (100), 204 (90); IR (KBr): υ (cm^−1^) 3429, 3306 (NH), 3031 (CH-aromatic), 2985 (CH-aliphatic), 1704, 1686 (C=O).

#### 2.2.2 Ethyl 2-amino-4-(3-hydroxyphenyl)-7-methyl-5-oxo-4,5-dihydropyrano[4,3-b]pyran-3-carboxylate (**4b**)

The compound was obtained as cream crystals with a yield of 67.7 % and had a melting point of 158 °C; *Rf* = 0.25 (EtOH/CHCl_3_ 1:9, v/v); ^1^H NMR (DMSO‑*d*_6_, 250 MHz) *δ*_H_ (ppm): 1.10 (t, 3H, *J* = 5.75 Hz, CH_2_–CH_3_), 2.21 (s, 3H, C_7_–CH_3_), 3.94–4.00 (app. m, 2H, CH_2_–CH_3_), 4.46 (s, 1H, C_4_–H), 6.28 (s, 1H, C_8_–H), 6.51–6.61 (app. m, 3H, phenyl-H_2,4,6_), 7.01 (t, 1H, *J* = 6.75 Hz, phenyl-H_5_), 7.69 (s, 2H, NH_2_), 9.24 (s, 1H, phenyl-OH); ^13^C NMR (DMSO‑*d*_6_, 75 MHz) *δ*_C_ (ppm): 14.63, 19.74, 34.64, 59.46, 77.64, 98.39, 104.20, 113.73, 115.19, 118.97, 129.19, 147.17, 157.39, 158.35, 159.20, 162.20, 162.84, 168.20; (EI) *m/z* (%): 343 (M^+^, 20), 270 (11), 250 (100), 204 (80); IR (KBr): υ (cm^−1^) 3442, 3328 (NH), 3400-3300 (OH), 3093 (CH-aromatic), 2986 (CH-aliphatic), 1706, 1677 (C=O).

#### 2.2.3 Ethyl 2-amino-4-(2,4-dichlorophenyl)-7-methyl-5-oxo-4,5-dihydropyrano[4,3-b]pyran-3-carboxylate (**4c**)

The compound was obtained as white crystals with a yield of 62.3 % and had a melting point range of 202–205 °C; *Rf* = 0.50 (EtOH/CHCl_3_ 1:9, v/v); ^1^H NMR (DMSO‑*d*_6_, 300 MHz) *δ*_H_ (ppm): 1.03 (t, 3H, *J* = 4.2 Hz, CH_2_–CH_3_), 2.21 (s, 3H, C_7_–CH_3_), 3.61 (q, 2H, *J* = 4.2 Hz, CH_2_–CH_3_), 4.88 (s, 1H, C_4_–H), 6.27 (s, 1H, C_8_–H), 7.25 (d, 1H, *J* = 5.1 Hz, phenyl-H_6_), 7.29–7.31 (dd, 1H, *J* = 5.1,1.2 Hz, phenyl-H_5_), 7.41 (d, 1H, *J* = 1.2 Hz, phenyl-H_3_), 7.83 (s, 2H, NH_2_); ^13^C NMR (DMSO‑*d*_6_, 125 MHz) *δ*_C_ (ppm): 14.55, 19.75, 33.21, 59.46, 75.74, 98.20, 101.76, 127.26, 128.96, 131.80, 133.77, 13.32, 141.47, 158.68, 159.30, 161.77, 163.38, 168.13; (EI) *m/z* (%):399 (M+4, 5), 397 (M+2, 9), 395 (M^+^, 14), 322 (12), 250 (100), 204 (90), 162 (15), 85 (12), 43 (40); IR (KBr): υ (cm^−1^) 3436, 3299 (NH), 3065 (CH-aromatic), 2985 (CH-aliphatic), 1713, 1681 (C=O).

#### 2.2.4 Ethyl 2-amino-4-(3-chlorophenyl)-7-methyl-5-oxo-4,5-dihydropyrano[4,3-b]pyran-3-carboxylate (**4d**)

The compound was obtained as cream crystals with a yield of 48.2 % and had a melting point range of 150–154 °C; *Rf* = 0.46 (EtOH/CHCl_3_ 1:9, v/v); ^1^H NMR (DMSO‑*d*_6_, 250 MHz) *δ*_H_ (ppm): 1.07 (t, 3H, *J* = 6 Hz, CH_2_–CH_3_), 2.22 (s, 3H, C_7_–CH_3_), 3.94–3.97 (app. m, 2H, CH_2_–CH_3_), 4.51 (s, 1H, C_4_–H), 6.31 (s, 1H, C_8_–H), 7.10–7.28 (m, 4H, phenyl-H_2, 4, 5, 6_), 7.79 (s, 2H, NH_2_); ^13^C NMR (DMSO‑*d*_6_, 75 MHz) *δ*_C_ (ppm): 14.54, 19.78, 34.95, 59.51, 76.83, 98.43, 103.16, 126.73, 126.97, 128.37, 130.33, 132.75, 148.25, 158.54, 159.14, 162.15, 163.33, 167.96; (EI) *m/z* (%):363 (M+2, 5), 361 (M^+^, 14), 288 (12), 250 (100), 204 (87), 162 (15), 85 (12), 43 (43); IR (KBr): υ (cm^−1^) 3427, 3295 (NH), 3094 (CH-aromatic), 2980 (CH-aliphatic), 1687 (C=O).

#### 2.2.5 Ethyl 2-amino-4-(4-cyanophenyl)-7-methyl-5-oxo-*)-7-methyl-5-oxo-4,5-dihydropyrano[4,3-b]pyran-3-carboxylate (**4e)***

The compound was obtained as white crystals with a yield of 45.1 % and had a melting point range of 235-239 °C; *R f*= 0.36 (EtOH/CHCl_3_ 1:9, v/v); ^1^H NMR (DMSO-*d_6_*, 250 MHz) *δ_H_* (ppm): 1.04 (t, 3H, *J*=5.75 Hz, CH_2_-CH_3_), 2.21 (s, 3H, C_7_-CH_3_), 3.94 (q, 2H, *J*=6.9 Hz, CH_2_-CH_3_), 4.58 (s, 1H, C_4_-H), 6.31 (s, 1H, C_8_-H), 7.37 (d, 2H,*J*=6.5 Hz, phenyl-H_3, 5_), 7.71 (d, 2H,*J*=6.5 Hz, phenyl-H_2_, _6_) 7.83 (s, 2H, NH_2_); ^13^C NMR (DMSO‑*d*_6_, 75 MHz) *δ*_C_ (ppm): 14.54, 19.77, 35.47, 59.54, 76.42, 98.42, 102.76, 109.52, 119.37, 129.57, 132.34, 151.37, 158.64, 159.19, 162.09, 163.51, 167.86; (EI) *m/z* (%):352 (M^+^, 18), 279 (23), 250 (100), 204 (87); IR (KBr): υ (cm^−1^) 3431, 3299 (NH), 3106 (CH-aromatic), 2976 (CH-aliphatic), 2229 (C ≡N), 1727 (C=O).

#### 2.2.6 Ethyl 2-amino-4-(4-bromophenyl)-7-methyl-5-oxo-4,5-dihydropyrano[4,3-b]pyran-3-carboxylate (**4f**)

The compound was obtained as cream crystals with a yield of 78.8 % and had a melting point range of 144–146 °C; *Rf* = 0.57 (EtOH/CHCl_3_ 1:9, v/v); ^1^H NMR (DMSO‑*d*_6_, 250 MHz) *δ*_H_ (ppm): 1.06 (t, 3H, *J* = 6 Hz, CH_2_–CH_3_), 2.21 (s, 3H, C_7_–CH_3_), 3.91–3.98 (app. m, 2H, CH_2_–CH_3_), 4.50 (s, 1H, C_4_–H), 6.30 (s, 1H, C_8_–H), 7.13 (d, 2H, *J* = 6.75 Hz, phenyl-H_2, 6_), 7.42 (d, 2H, *J* = 6.75 Hz, phenyl-H_3, 5_) 7.77 (s, 2H, NH_2_); ^13^C NMR (DMSO‑*d*_6_, 75 MHz) *δ*_C_ (ppm): 14.60, 19.76, 34.64, 59.51, 76.96, 98.40, 103.41, 119.71, 130.62, 131.18, 145.23, 158.39, 159.14, 162.13, 163.17, 168.02; (EI) *m/z* (%):407 (M+2, 11), 405 (M^+^, 12), 332 (11), 334 (10), 250 (100), 204 (98); IR (KBr): υ (cm^−1^) 3462, 3305 (NH), 3064 (CH-aromatic), 2980 (CH-aliphatic), 1731, 1690 (C=O).

#### 2.2.7 Ethyl 2-amino-7-methyl-4-(4-nitrophenyl)-5-oxo-4,5-dihydropyrano[4,3-b]pyran-3-carboxylate (**4g**)

The compound was obtained as cream crystals with a yield of 51.3 % and had a melting point range of 160.162 °C; *Rf* = 0.43 (EtOH/CHCl_3_ 1:9, v/v); ^1^H NMR (DMSO‑*d*_6_, 250 MHz) *δ*_H_ (ppm): 1.04 (t, 3H, *J* = 5.75 Hz, CH_2_–CH_3_), 2.21 (s, 3H, C_7_–CH_3_), 3.90–3.97 (app. m, 2H, CH_2_–CH_3_), 4.64 (s, 1H, C_4_–H), 6.32 (s, 1H, C_8_–H), 7.45 (d, 2H, *J* = 7.25 Hz, phenyl-H_2, 6_), 7.85 (s, 2H, NH_2_), 8.12 (d, 2H, *J* = 7.25 Hz, phenyl-H_3, 5_); ^13^C NMR (DMSO‑*d*_6_*,* 75 MHz) *δ*_C_ (ppm): 14.54, 19.77, 35.31, 59.59, 76.30, 98.43, 102.63, 123.60, 129.80, 146.43, 153.44, 158.68, 159.16, 162.08, 163.61, 167.84; (EI) *m/z* (%):372 (M^+^, 11), 299 (15), 250 (100), 204 (83); IR (KBr): υ (cm^−1^) 3470, 3318 (NH), 3078 (CH-aromatic), 2979 (CH-aliphatic), 1726, 1687 (C=O), 1509, 1345 (NO_2_).

#### 2.2.8 Ethyl 2-amino-7-methyl-5-oxo-4-(4-(trifluoromethyl)phenyl)-4,5-dihydropyrano[4,3-b]pyran-3-carboxylate (**4h**)

The compound was obtained as yellow crystals with a yield of 34.2 % and had a melting point range of 155–158 °C; *Rf* = 0.50 (EtOH/CHCl_3_ 1:9, v/v); ^1^H NMR (DMSO‑*d*_6_, 250 MHz) *δ*_H_ (ppm): 1.04 (t, 3H, *J* = 5.75 Hz, CH_2_–CH_3_), 2.21 (s, 3H, C_7_–CH_3_), 3.91–3.97 (app. m, 2H, CH_2_–CH_3_), 4.61 (s, 1H, C_4_–H), 6.32 (s, 1H, C_8_–H), 7.40 (d, 2H, *J* = 6.75 Hz, phenyl-H_2, 6_), 7.60 (d, 2H, *J* = 6.75 Hz, phenyl-H_3, 5_), 7.81 (s, 2H, NH_2_); ^13^C NMR (DMSO‑*d*_6_, 75 MHz) *δ*_C_ (ppm): 14.52, 19.75, 35.15, 59.51, 76.71, 98.40, 103.11, 122.98, 125.21, 127.19, 129.25, 150.42, 158.57, 159.19, 162.12, 163.39, 167.94; (EI) *m/z* (%):395 (M^+^, 13), 322 (14), 250 (100), 204 (95); IR (KBr): υ (cm^−1^) 3439, 3307 (NH), 3071 (CH-aromatic), 2986 (CH-aliphatic), 1714, 1684 (C=O).

#### 2.2.9 Ethyl 2-amino-4-(4-chlorophenyl)-7-methyl-5-oxo-4,5-dihydropyrano[4,3-b]pyran-3-carboxylate (**4i**)

The compound was obtained as cream crystals with a yield of 24.8 % and had a melting point range of 147–150 °C; *Rf* = 0.47 (EtOH/CHCl_3_ 1:9, v/v); ^1^H NMR (DMSO‑*d*_6_*,* 250 MHz) *δ*_H_ (ppm): 1.06 (t, 3H, *J* = 6 Hz, CH_2_–CH_3_), 2.21 (s, 3H, C_7_–CH_3_), 3.91–3.98 (app. m, 2H, CH_2_–CH_3_), 4.51 (s, 1H, C_4_–H), 6.30 (s, 1H, C_8_–H), 7.18 (d, 2H, *J* = 7 Hz, phenyl-H_2, 6_), 7.29 (d, 2H, *J* = 7 Hz, phenyl-H_3, 5_),7.77 (s, 2H, NH_2_); ^13^C NMR (DMSO‑*d*_6_, 75 MHz) *δ*_C_ (ppm): 14.59, 19.76, 34.55, 59.50, 77.02, 98.41, 103.46, 128.27, 130.21, 131.21, 144.80, 158.39, 159.14, 162.13, 163.18, 168.02; (EI) *m/z* (%): 363 (M+2, 4), 361 (M^+^, 12), 289 (18), 250 (100), 204 (60), 162 (14), 151 (15), 85 (18), 43 (35); IR (KBr): υ (cm^−1^) 3470, 3308 (NH), 2982 (CH-aromatic), 2933 (CH-aliphatic), 1724, 1690 (C=O).

#### 2.2.10 Ethyl 2-amino-7-methyl-5-oxo-4-(3,4,5-trimethoxyphenyl)-4,5-dihydropyrano[4,3-b]pyran-3-carboxylate (**4j**)

The compound was obtained as white crystals with a yield of 54.7 % and had a melting point range of 197–200 °C; *Rf* = 0.37 (EtOH/CHCl_3_ 1:9, v/v); ^1^H NMR (DMSO‑*d*_6_, 250 MHz) *δ*_H_ (ppm): 1.13 (t, 3H, *J* = 5.75 Hz, CH_2_–CH_3_), 2.22 (s, 3H, C_7_–CH_3_), 3.60 (s, 3H, phenyl-OCH_3_), 3.70 (s, 6H, phenyl-OCH_3_), 4.00 (q, 2H, *J* = 5.75 Hz*,* CH_2_–CH_3_), 4.52 (s, 1H, C_4_–H), 6.31 (s, 1H, C_8_–H), 6.42 (s, 2H, phenyl-H_2, 6_), 7.72 (s, 2H, NH_2_); ^13^C NMR (DMSO‑*d*_6_, 75 MHz) *δ*_C_ (ppm): 14.73, 19.78, 35.01, 56.19, 59.50, 60.37, 77.36, 98.44, 103.90, 105.44, 136.58, 141.50, 152.83, 158.50, 159.30, 162.29, 162.95, 168.19; (EI) *m/z* (%): 417 (M^+^, 33), 340 (22), 291 (19), 250 (100), 204 (62); IR (KBr): υ (cm^−1^) 3422, 3305 (NH), 3093 (CH-aromatic), 2992 (CH-aliphatic), 1704 (C=O).

### 2.3 *In vitro* anti-proliferative activity evaluation

Two cancer cell lines, including the human colorectal (SW-480) and the human breast (MCF-7) cells, were taken from the National Cell Bank of Iran (NCBI, Pasteur Institute, Tehran, Iran). All cells were cultured in RPMI-1640 medium containing 10 % fetal bovine serum (FBS) (Gibco Invitrogen Co., Scotland, UK), antibiotics (penicillin and streptomycin, 10 % v/v), at 37 °C in a humid atmosphere containing 5 % CO_2_. All designed compounds (**4a**-**j**) were evaluated by the standard 3-(4,5-dimethylthiazol-yl)-2,5-diphenyl-tetrazolium bromide (MTT) assay to check their anti-cancer activity. By using trypsin/EDTA 0.5 % solution, the cells were harvested and then seeded in 96-well microplates at a density of 1 × 10^4^ and 8 × 10^3^ cells per well, respectively, for MCF-7 and SW480 cell lines in 100 μL of complete culture medium. After 24 h of incubation, the cells were treated with five different concentrations (1–500 μM) of cisplatin (as the positive control) and the synthesized derivatives in a triplicate manner. After 72 h of incubation, formazan crystals were obtained by replacing the media with 100 μL fresh MTT solution. The plates were incubated for 4 h at 37 °C. To dissolve the formazan crystals, the media was removed, 150 μL of DMSO was added, and the cells were incubated at 37 °C in the dark for 10 min. Finally, a microplate ELISA reader was applied to record the absorbance of each well at 490 nm. To analyze the data, Excel 2016 and Curve Expert 1.4 [26] were used. The data were provided in terms of mean IC_50_ value ± SD [27].

### 2.4 Free radical-scavenging activity evaluation

Radical-scavenging activity was determined by the 2,2-Diphenyl-1-picrylhydrazyl (DPPH) assay as described previously [12,28,29]. A mixture of different concentrations of the compounds and DPPH methanolic solution (110 μM) was shaken in the dark at room temperature for 30 min. The mixture absorbance was measured at 517 nm. Quercetin was used as the positive control. After calculating the percentage of scavenging activities by the following equation, the EC_50_ values were obtained from linear regression plots between the concentrations of the tested compounds and the percentage of the radical-scavenging activities. Each concentration was analyzed in three independent experiments conducted in triplicates.

Radical scavenging activity (%) = 100 × (Abs_control_−Abs_compound_)∕Abs_control_ [12].

### 2.5 Molecular docking study

Docking was performed by AutoDock 4.2 and AutoDock Tools 1.5.4 (ADT) [30]. The X-ray crystal structure of CDK2 with 4-(3-hydroxyanilino)-6,7-dimethoxyquinazoline as the co-crystallized ligand (PDB ID: 1DI8) was taken from the Protein Data Bank (http://www.rcsb.org). Before docking, the innate ligand and water molecules were removed from 1DI8, then hydrogens were added, and nonpolar hydrogens were merged. Finally, Gasteiger charges were calculated for protein 1DI8. 3D structures of the ligands were sketched and minimized using Avogadro software [31]. PDBQT formats of the ligands were prepared by adding Gasteiger charges and setting the degree of torsions. The grid maps were constructed by considering a grid box of 50 × 50 × 50 dimensions with 0.375 Å spacing centered at x = −9.052, y = 48.898, and z = 11.469 Å. For the docking process, the macromolecule was considered to be rigid. Lamarckian genetic search algorithm was chosen, and the number of runs was set at 70. The validity of the docking procedure was confirmed using a co-crystallized inhibitor as the ligand and the above-mentioned protocol.

### 2.6 Molecular dynamics simulation

Molecular dynamics (MD) simulation analysis was performed to explore the dynamic behavior of the complexes. GROMACS 2016.3 software was utilized for simulations, and the CHARMM27 force field was employed to represent molecular interactions accurately [32]. Ligand parameters were generated using the SwissParam web server. The system was solvated in a cubic periodic box, and counter ions were added for system neutralization. Following energy minimization, the system underwent equilibration in two successive phases: NVT (constant number of particles, volume, and temperature) and NPT (constant number of particles, pressure, and temperature). Subsequently, a production MD run was conducted with analyses based on a total of 100 ns MD trajectories. Root-mean-square deviation (RMSD), root-mean-square fluctuation (RMSF), and hydrogen bond analyses were performed to assess ligand stability and flexibility within the binding pocket over the simulation period. The molecular mechanics Poisson Boltzmann surface area (MM-PBSA) method was utilized to estimate binding free energies. All graphical representations were generated using Excel software.

### 2.7 Prediction of drug-likeness and pharmacokinetic properties

Drug-likeness profiling, GI absorption, and P-gp substrate potency of the synthesized compounds were predicted by the SwissADME (http://www.swissadme.ch) free web tool. The *in-silico* prediction of P-gp inhibitory activity, Blood-brain barrier (BBB) permeation, Caco 2 cell permeability, and MDCK cell permeability were carried out using preADMET (http://preadmet.qsarhub.com).

### 2.8 DFT analysis

The density functional theory (DFT) was carried out on Gaussian 09 at the B3LYP function coupled with the 6–311++G (d,p) basis set. The structural geometries were optimized at their ground state energies. Subsequently, energies of the highest occupied molecular orbitals (HOMO), lowest unoccupied molecular orbitals (LUMO), and energy gap between HOMO and LUMO were obtained for **4g**, **4i**, **4j**, and **4d**. Moreover, thermochemical parameters were determined by using HOMO and LUMO energies. The Theoretical FT-IR spectra were also calculated at their optimized geometries through DFT/6–311++G (d,p) functions.

## 3 Results and discussion

### 3.1 Synthesis

**Scheme 1:**
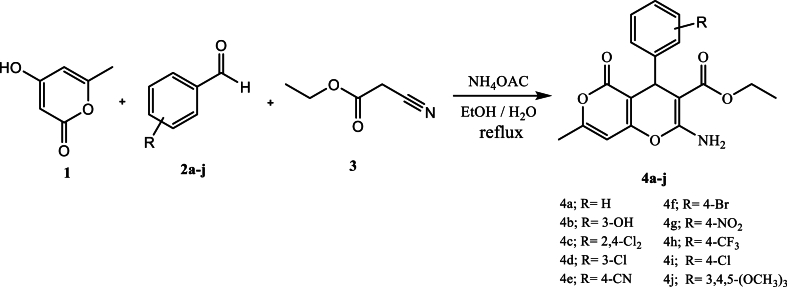
Synthetic route for the preparation of 5-oxo-4,5-dihydropyrano[4,3-*b*]pyran derivatives.

The general procedure for the synthesis of the target dihydropyranopyran derivatives is shown in Scheme 1.A mixture of 4-hydroxy-6-methyl-2-pyrone (**1**), corresponding benzaldehyde (**2a-j**), and ethyl cyanoacetate (**3**) in the presence of ammonium acetate was refluxed in water and ethanol for 24 h. The final pure products (**4a-j**) were obtained after washing with a suitable solvent or, in some cases, after applying TLC. The structures of the synthesized compounds were confirmed by ^1^H NMR, ^13^C NMR, MS, and FT-IR analyses.

### 3.2 Anti-proliferative activity of the synthesized compounds

The anti-proliferative activity of all the compounds was investigated against SW-480 and MCF-7 cancer cell lines by using MTT assay. Cisplatin was used as the positive control. The concentration required to inhibit the cancer cells' growth by 50 % (IC_50_) was estimated for each derivative (Table 1). Compounds **4g**, **4i**, and **4j** (bearing 4-NO_2_, 4-Cl, and 3,4,5-(OCH_3_)_3_, respectively) with IC_50_ values of 34.6, 35.9, and 38.6 μM, respectively, against SW-480 cell line, and 42.6, 34.2, and 26.6 μM, against MCF-7 cells showed the highest anti-proliferative effects. Compounds **4a**, **4d**, and **4h** had the lowest anti-proliferative activities against SW-480 with IC_50_ values of 90.5, 87.2, and 67.2 μM, respectively. Derivatives **4d** and **4e** were the least active compounds against MCF-7 cells with IC_50_ values of 104.9 and 71.0 μM. The rest of the compounds exhibited moderate anti-proliferative activities with an IC_50_ range of 41.2–54.3 μM.

**Table 1:** Chemical structures, anti-proliferative, and radical scavenging activities of the synthesized compounds.


Compound	R	Cytotoxicity IC_50_ (μM)^a^		DPPH Radical scavenging EC_50_ (μM)^a^
		**SW-480**	**MCF-7**	
**4a**	H	90.5 ± 1.8	54.3 ± 5.8	>1000
**4b**	3-OH	48.8 ± 8.5	45.5 ± 4.9	644 ± 2.8
**4c**	2,4-(Cl)_2_	41.2 ± 0.2	48.2 ± 3.9	>1000
**4d**	3-Cl	87.2 ± 2.5	104.9 ± 1.3	>1000
**4e**	4-CN	45.3 ± 5.5	71.0 ± 4.5	832 ± 5.1
**4f**	4-Br	49.6 ± 1.5	54.2 ± 5.1	>1000
**4g**	4-NO_2_	34.6 ± 1.4	42.6 ± 0.1	>1000
**4h**	4-CF_3_	67.2 ± 7.4	51.0 ± 5.9	>1000
**4i**	4-Cl	35.9 ± 4.2	34.2 ± 3.6	>1000
**4j**	3,4,5-(OCH_3_)_3_	38.6 ± 5.0	26.6 ± 3.0	580 ± 3.5
**Cisplatin**	–	15.2 ± 0.35	9.9 ± 0.14	–
**Quercetin**	–	–	–	9.8 ± 0.5

a Values represent means ± SD of three independent experiments.

According to the results, a brief structure-activity relationship (SAR) can be proposed. It seems that substitution on the C_4_-phenyl ring improved the anti-proliferative activity of the ethyl 2-amino-7-methyl-5-oxo-4,5-dihydropyrano[4,3-*b*]pyran-3-carboxylate derivatives; as most of the compounds had superior activities to derivative **4a**. Exceptions were **4d**, **4e**, and **4h** (bearing 3-Cl, 4-CN, and 4-Br moieties on the phenyl ring, respectively), which showed IC_50_ values higher than **4a**. Placing an electron-withdrawing chlorine atom at the *meta* position of the phenyl ring (as in **4d**) diminished the activity; however, substituting electron-donating groups at this position (as in **4b** bearing 3-OH substitution) enhanced the activity. Accordingly, introducing three electron-donating methoxy groups at the 3, 4, and 5 positions of the phenyl ring caused compound **4j** to be one of the most potent derivatives. The nature of the substituents introduced to the *para* position of the C_4_-phenyl had a considerable effect on the anti-proliferative potency of the dihydropyranopyran derivatives. It seems that the insertion of electron-withdrawing moieties at this position would reduce activity. Compound **4i** showed better activity than **4h** and **4e** (bearing strong electron-withdrawing 4-CN and 4-CF_3_ groups) due to the reduced electron-withdrawing capacity of the chlorine atom at the *para* position. In compound **4g**, the insertion of a nitro group at the *para* position as a hydrogen bond acceptor moiety, led to a noticeable increase in activity compared with **4h** and **4e**.

### 3.3 Free radical scavenging activity

The derivatives were evaluated for their radical scavenging potency against DPPH. The calculated EC_50_ values are listed in Table 1. Compounds **4b**, **4e**, and **4j**, bearing 3-hydroxyphenyl, 4-cyanophenyl, and 3,4,5-trimethoxyphenyl substitutions, showed radical scavenging activity with EC_5__0_ values of 644, 832, and 580 μM, respectively. Compound **4j**, bearing 3,4,5-trimethoxy substitutions on the C_4_-phenyl, was the most potent radical scavenger.

### 3.4 Molecular docking analysis

Molecular docking is a widely used technique to assess *in-silico* the affinity and inhibitory potential of compounds showing anti-cancer activity against a specific target [27,33,34]. Cyclin-dependent kinases (CDKs) are a family of proteins that contribute to cell proliferation by regulating the progression of cell cycle and transcription [35,36]. A literature review revealed that CKD2 can be proposed as a valid target for the anti-cancer pyran derivatives [[21], [22], [23], [24]]. CKD inhibitors compete with ATP to bind at the kinase site, causing suppression of CDK hyperactivation through inhibition of kinase phosphorylation and, consequently, preventing extreme cell proliferation [37]. Therefore, molecular docking analysis was carried out to predict the binding affinity and interactions of the dihydropyranopyran derivatives in this study (**4a**-**j**) into the ATP binding site of CDK2.

**Fig. 2:**
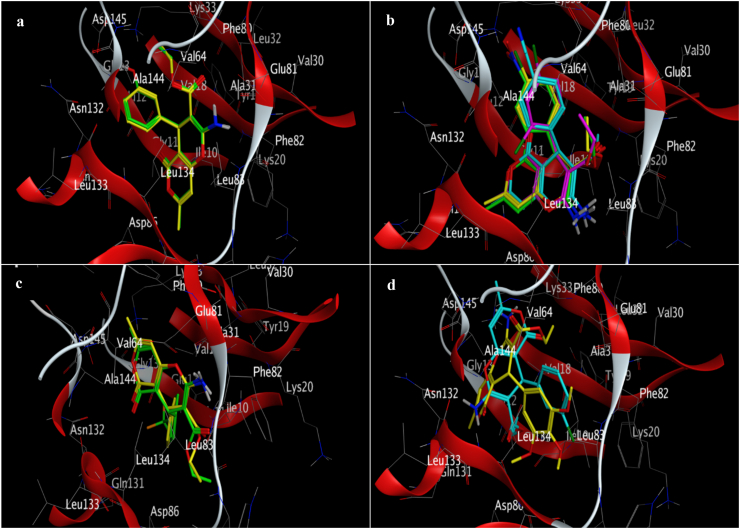
Representation of different binding modes of the target compounds (**4a**-**j**) in the active site of CDK2. **(a) 4a** (yellow) and **4b** (green). **(b) 4i** (yellow), **4g** (green), **4c** (purple), and **4e** (cyan). **(c) 4h** (green) and **4f** (yellow). **(d) 4j** (yellow) and **4d** (cyan). (For interpretation of the references to color in this figure legend, the reader is referred to the Web version of this article.)

**Fig. 3:**
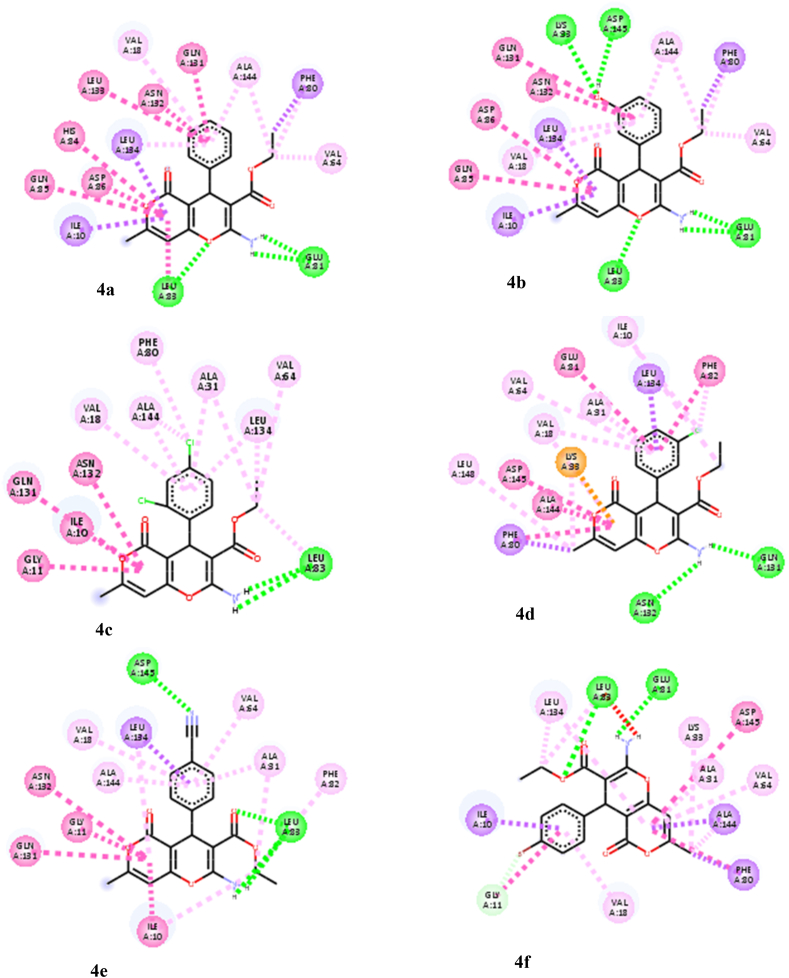

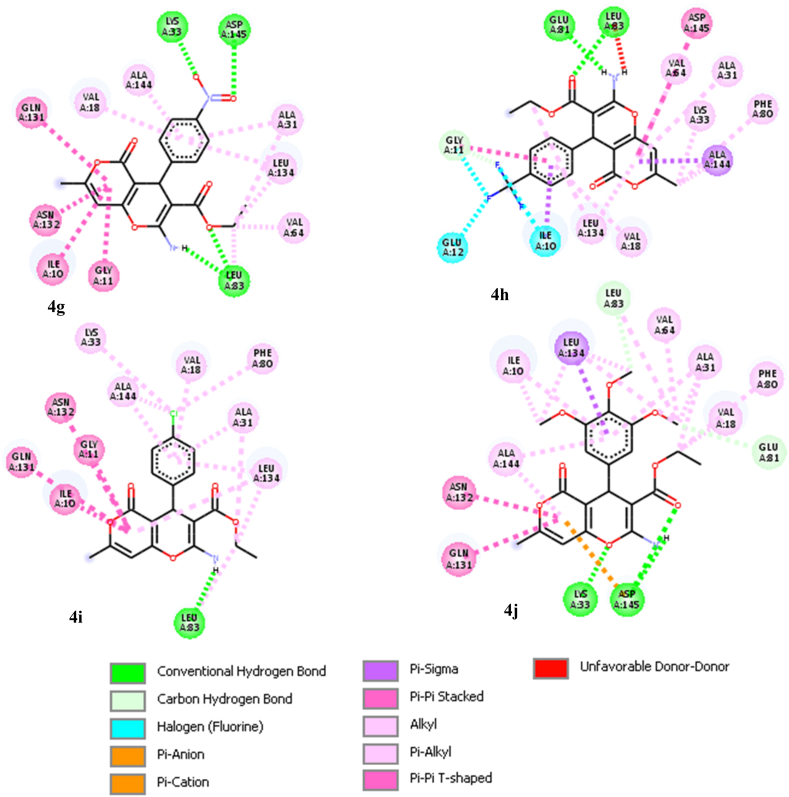
Interactions of the target compounds (**4a-j**) in the binding site of CDK2.

The docking procedure was initially validated using a self-docking approach. The co-crystallized ligand (DTQ) was re-docked into the binding site of CDK2 (PDB code: 1DI8). The RMSD between the best pose of the co-crystallized ligand docked into the binding site of CDK2 and the one in the crystal structure was found 1.46 Å. This confirmed the validity of the docking procedure. The calculated binding free energies (ΔG) and interaction details for the target compounds (**4a**-**j**), DTQ and BMS-265246 (a well-known CDK2 inhibitor), are presented in Table 2. All derivatives were well accommodated within the active site of CDK2 with binding energy values ranging from −7.52 to −8.72 kcal/mol, which was comparable to the binding energies of DTQ (−8.00 kcal/mol) and BMS-265246 (−7.76 kcal/mol). The 3D binding orientations of the derivatives are illustrated in Fig. 2. The compounds had different binding modes. It can be concluded that the nature and position of the substitutions on the C_4_-phenyl ring determined the orientations of the derivatives in the binding site of CDK2. The ligand–protein interactions are depicted in Fig. 3. As shown in Fig. 2a, compounds **4a** and **4b** had the same binding orientations. The NH_2_ moieties established two hydrogen bonds with GLU81 residue, and the oxygen atom in the dihydropyran part of the compounds made an additional hydrogen bonding interaction with LEU83. The pyranon nucleus formed hydrophobic interactions with LEU134, ILE10, GLN85, and ASP86 amino acids, while the C_4_-phenyl ring contributed to establishing hydrophobic interactions with ALA144, ASN132, GLN131, LEU133, and VAL18 residues. Moreover, the ethyl of carboxylate groups occupied the hydrophobic pocket surrounded by PHE80, ALA144, and VAL64 residues. In the case of compound **4b**, the 3-OH group provided two additional hydrogen bonding interactions with LYS33 and ASP145. According to the docking results, **4i**, **4g**, **4c**, and **4e** were oriented similarly in the ATP binding site of CDK2 (Fig. 2b). The pyranon ring contributed to the formation of hydrophobic interactions with ILE10, GLY11, GLN131, ASN132, and LEU134 residues. The phenyl ring was surrounded by lipophilic residues, including ALA144, LEU134, ALA31, and VAL18. A key hydrogen bonding interaction between NH_2_ and LEU83 was observed in the mentioned compounds. The **4e** and **4g** derivatives made another hydrogen bond with LEU83 through the carboxylate substitution. Moreover, **4e** and **4g** formed a hydrogen bond with amino acid residue ASP145 through the phenyl ring substitution (4-CN and 4-NO_2_). Compound **4g** formed a more stable complex *via* establishing an additional hydrogen bond with LYS33 through the 4-NO_2_ substitution. In compounds **4f** and **4h** (Fig. 2c), carboxylate and amino substitutions were involved in hydrogen bonding interactions with LEU83 and GLU81, respectively. The pyranon and its methyl substitution occupied the hydrophobic pocket comprising PHE80, ALA144, ALA31, VAL64, LEU134, LYS33, and PHE80. The phenyl ring contributed to hydrophobic interactions with the nearby residues, including ILE10, GLY11, and VAL18. Furthermore, the 4-CF_3_ substitution in compound **4h** was involved in halogen bonding interaction with ILE10, GLY11, and GLU12 residues. Compounds **4d** and **4j** were oriented differently in the CDK2 active site (Fig. 2d). In the case of compound **4d**, two hydrogen bonds were observed between the amino group and the two residues, ASN132 and GLN131. The phenyl ring showed lipophilic interaction with VAL18, ALA31, VAL64, GLU81, and PHE82, while the pyranon moiety was accommodated in the pocket comprising ALA144, LYS33, VAL18, PHE80, LEU148, and ASP145 amino acids through hydrophobic interactions. 3-Cl and ethyl carboxylate groups established hydrophobic interaction with ILE10. Finally, compound **4j** made a stable ligand-CDK2 complex by establishing hydrogen bonding, electrostatic, and hydrophobic interactions. This derivative formed three hydrogen bonds; with amino acid residue ASP145 through its amino and carboxylate groups and amino acid LYS33 through the oxygen atom of the dihydropyran ring. The pyranon ring contributed to an electrostatic interaction with ASP145 as well as some hydrophobic interactions with ALA144, GLN131, and ASN132. The trimethoxyphenyl ring was surrounded by hydrophobic residues including VAL64, LEU83, LEU134, ALA31, ALA144, and ILE10.

**Table 2:** Docking results of the target derivatives (**4a-j**) within the binding site of CDK2.

Compound	ΔG (kcal/mol)	Interactions	Atom of ligand	Amino acid (distance; Å)
				
**4a**	−8.58	H-bonding	O of dihydropyran	LEU83 (2.28)
		H-bonding	NH_2_	GLU81 (1.97, 2.17)
		Hydrophobic	Phenyl	ALA144 (3.6), ASN132(5.60), GLN131 (5.59), LEU133 (4.93), VAL18 (5.33)
		Hydrophobic	Pyranon	LEU134 (3.37), ILE10 (3.94), HIS84(5.58), GLN85 (6.01), ASP86 (6.15)
		Hydrophobic	CH_3_–CH_2_–COO	PHE80 (3.66)
		Hydrophobic	CH_3_–CH_2_–COO	PHE80 (3.95), ALA144 (3.26), VAL64 (3.50)
**4b**	−8.72	H-bonding	NH_2_	GLU81 (2.00,2.18)
		H-bonding	3-OH	LYS33 (2.53), ASP145 (2.16)
		H-bonding	O of dihydropyran	LEU83 (2.34)
		Hydrophobic	Phenyl	ALA144 (3.64), ASN132(5.86), GLN131 (6.02), LEU134 (5.01), VAL18 (5.27)
		Hydrophobic	Pyranon	LEU134 (3.50), ILE10 (3.79), GLN85 (5.72), ASP86 (5.68)
		Hydrophobic	CH_3_–CH_2_–COO	PHE80 (3.83)
		Hydrophobic	CH_3_–CH_2_–COO	PHE80 (3.79), ALA144 (3.43), VAL64 (3.79)
**4c**	−7.52	H-bonding	NH_2_	LEU83 (2.65, 3.03)
		Hydrophobic	Phenyl	VAL18 (4.46), ALA31 (4.52), LEU134 (5.34), ALA144 (4.28)
		Hydrophobic	Pyranon	ILE10 (5.0), GLY11 (5.69), GLN131 (5.94), ASN132(5.72)
		Hydrophobic	4-Cl	PHE80(3.35), ALA144 (3.84)
		Hydrophobic	CH_3_–CH_2_–COO	LEU83 (3.95), LEU134 (4.31), VAL64 (4.42), ALA31 (4.39)
**4d**	−8.37	H-bonding	NH_2_	ASN132(2.21), GLN131 (1.96)
		Hydrophobic	CH_3_ on pyranon	LEU148 (4.21), LYS33 (4.85)
		Hydrophobic	3-Cl	PHE82(4.11), ILE10 (4.75)
		Hydrophobic	CH_3_–CH_2_–COO	ILE10 (4.96)
		Hydrophobic	Phenyl	VAL18 (5.82), ALA31 (3.65), VAL64 (5.49), GLU81 (5.69), PHE82(6.04), LEU134 (3.51)
		
		Electrostatic Hydrophobic	Pyranon Pyranon	LYS33(4.49) ALA144 (4.07), LYS33 (4.80), VAL18 (4.93), PHE80 (4.60), LEU148 (4.21), ASP145 (5.75)
**4e**	−8.31	H-bonding	NH_2_	LEU83 (2.22, 2.29)
		H-bonding	CH_3_–CH_2_–COO	LEU83 (1.92)
		H-bonding	4-CN	ASP145 (2.42)
		Hydrophobic	Phenyl	VAL18 (4.95), ALA31 (4.80), ALA144 (3.81), VAL64 (5.16), LEU134 (3.87)
		Hydrophobic	Pyranon	ILE10 (5.04), GLY11 (5.62), GLN131 (5.97), ASN132(6.07), LEU134 (5.37)
		Hydrophobic	CH_3_–CH_2_–COO	PHE82(4.14), ALA3194.52), ILE10 (3.70)
**4f**	−8.16	H-bonding	NH_2_	GLU81 (1.97)
		H-bonding	CH_3_–CH_2_–COO	LEU83 (2.38)
		Hydrophobic	Pyranone	PHE80 (5.21), ALA144 (3.41), ALA31 (5.13), VAL64 (4.86), LEU134 (5.28)
		Hydrophobic	CH_3_–CH_2_–COO	LEU83 (4.68), LEU134 (3.58)
		Hydrophobic	CH_3_ on pyranon	ALA144 (4.21), LYS33 (5.0), PHE80 (3.24)
		Hydrophobic	Phenyl	ILE10 (3.32), VAL18 (4.62)
		Halogen	4-Br	GLY11 (3.45)
**4g**	−8.49	H-bonding	NH_2_	LEU83 (2.23)
		H-bonding	CH_3_–CH_2_–COO	LEU83 (1.71)
		H-bonding	NO_2_	LYS33(1.69), ASP145(2.09)
		Hydrophobic	CH_3_–CH_2_–COO	ALA31 (4.43), LEU83 (3.96), LEU134 (4.17), VAL64(4.58)
		Hydrophobic	Phenyl	VAL17(4.17), LEU134 (5.48), ALA31 (5.0), ALA144 (4.34)
		Hydrophobic	Pyranon	ILE10 (5.17), GLY11 (5.02), GLN131 (5.88), ASN132(4.19)
**4h**	−8.44	H-bonding	NH_2_	GLU81 (1.88)
		H-bonding	CH_3_–CH_2_–COO	LEU83 (2.02)
		Hydrophobic	CH_3_–CH_2_–COO	LEU134 (415)
		Hydrophobic	CH_3_ on pyranon	ALA144 (4.34), LYS33(4.49), PHE80 (4.19)
		Hydrophobic	Pyranon	LEU134 (5.15), VAL64 (5.20), ALA144 (3.44), ALA31 (5.65), PHE80 (5.64)
		Hydrophobic	Phenyl	ILE10 (3.13), GLY11 (5.69), VAL18 (4.38)
		Halogen	4-CF_3_	ILE10 (2.96), GLY11 (2.91), GLU12 (3.40)
**4i**	−8.01	H-bonding	NH_2_	LEU83 (2.36)
		Hydrophobic	CH_3_–CH_2_–COO	LEU134 (4.00), ALA31 (4.28), LEU83 (4.42)
		Hydrophobic	Phenyl	ALA144 (4.18), LEU134 (5.32), ALA31 (4.91), VAL18 (4.38)
		Hydrophobic	4-Cl	PHE80 (4.47), ALA144 (3.68), LYS33 (5.16)
		Hydrophobic	Pyranon	ILE10 (5.12), GLY11 (5.46), GLN131 (5.83), ASN132 (6.05)
**4j**	−8.50	H-bonding	NH_2_	ASP145 (2.69)
		H-bonding	O of dihydropyran	LYS33 (2.45)
		H-bonding	CH_3_–CH_2_–COO	ASP145 (2.54)
		Electrostatic	Pyranon	ASP145 (3.95)
		Hydrophobic	3-OCH_3_	VAL64 (4.86), LEU83 (4.41), LEU134 (4.61), ALA31 (3.89)
		Hydrophobic	4-OCH_3_	LEU134 (4.23)
		Hydrophobic	5-OCH_3_	LEU134 (5.42), ILE10 (4.09)
		Hydrophobic	CH_3_–CH_2_–COO	ALA31 (3.36), VAL18 (4.05), PHE80 (4.47)
		Hydrophobic Hydrophobic	Phenyl Pyranon	ALA31 (5.0), LEU134 (3.66), ALA144 (5.41), ILE10 (5.51) ALA144 (4.71), GLN131 (5.08), ASN132(5.16)
**DTQ**^a^	−8.00	H-bonding	N of quinazoline	LEU83 (2.08)
		H-bonding	OH	ASP145 (2.27)
		Hydrophobic	Pyrimidine of quinazoline	VAL64 (5.24),ALA31 (3.94), GLU81 (5.41), LEU83, LEU134 (3.12)
		Hydrophobic	Phenyl of quinazoline	LEU134 (3.18), ILE10 (5.18), HIS84 (5.91), PHE82(5.95)
		Hydrophobic	Aniline	ALA144 (3.83), LYS33 (5.03), VAL18 (5.29)
		Hydrophobic	7-OCH_3_	PHE82(5.16)
		Electrostatic	Aniline	ASP145 (4.56)
**BMS-265246**^b^	−7.76	H-bonding	NH of Pyrazole	LEU83 (2.20)
		H-bonding	N of pyridine	LEU83 (1.89)
		Hydrophobic	Pyrazole	ILE10 (5.05), LEU134 (3.74)
		Hydrophobic	Pyridine	LEU83 (5.60), ILE10 (5.77), LEU134 (3.79),LEU83 (5.60), ALA31 (3.92)
		Hydrophobic	Butyl	ALA144 (3.90)
		Hydrophobic	Aniline	ALA31 (5.05), LYS33 (5.27), VAL18 (5.14), ALA144 (4.11), VAL64 (5.09), PHE80 (3.87)
		Hydrophobic	Methyl	PHE80 (4.20), LYS33 (4.15), LEU148 (4.86)
		Halogen	2-F	ALA144 (3.40)
		Halogen	6-F	ALA31 (3.90)

a 4-(3-Hydroxyanilino)-6,7-dimethoxyquinazoline.

b (4-Butoxy-1*H*-pyrazolo [3,4-*b*]pyridin-5-yl) (2,6-difluoro-4-methylphenyl)methanone.

The docking results exhibited that stabilization of DTQ in the active site of CDK2 resulted from key hydrogen bonds with residues LEU83 and ASP145 at the distances of 2.08 and 2.28 Å, respectively, as well as an electrostatic interaction with ASP145 at a distance of 4.56 Å, and some hydrophobic interactions with ILE10, VAL18, ALA31, LYS33, AVAL64, GLU81, PHE82, LEU83, HIS84, LEU134, and ALA144. All the active derivatives established hydrogen bonds with one or both LEU83 and ASP145 residues *via* the amine group, the carboxylate moiety, the oxygen atom of the dihydropyran ring, or the substitutions on the C_4_-phenyl ring. The least potent compound **4d**, did not form any hydrogen bonding with the mentioned amino acids. Moreover, in the target compounds, ethyl of the carboxylate group, pyranon ring, and the C_4_-phenyl participated in establishing hydrophobic interactions with the same residues as DTQ did. The substitutions on the C_4_-phenyl ring had a key role in the orientations and interactions of the derivatives. For example, in the case of **4j**, the three methoxy substitutions on the C_4_-phenyl ring provided some strong hydrophobic interactions with VAL64, LEU83, LEU134, ALA31, and ILE10; while, in the case of compound **4g**, the nitro substitution formed tough hydrogen bonding interactions with LYS33 and ASP145.

### 3.5 MD simulations

To evaluate the stability of compound **4j** and DTQ within the CDK2 active site, MD simulations were conducted.

**Fig. 4:**
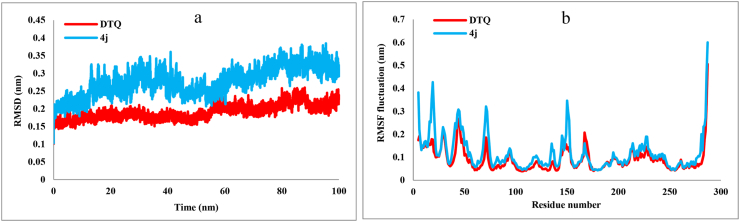
The RMSD (a) and RMSF (b) analyses results of DTQ-CDK2 and **4j-**CDK2 complexes over the MD run.

The RMSD analysis helped to study the degree of deviation from the initial structure during the simulation time. Fig. 4a presents the average backbone RMSD values for compound **4j** and DTQ complex structures over the 100 ns simulation period. Compound **4j** exhibited an average RMSD value of 2.5 Å, with fluctuations ranging from 1.0 to 3.7 Å. DTQ complex displayed a lower average RMSD value of 1.9 Å. Notably, fluctuations in the RMSD value for the DTQ complex remained within the range of 1.0–2.5 Å. The RMSF values explained the structural integrity of CDK2 and the flexibility of amino acid residues bound to compound **4j** and DTQ during the 100 ns MD simulation period (Fig. 4b). Significant fluctuations were observed in the amino acid residues located at the terminal end sections. Moreover, **4j** and DTQ complexes displayed a similar RMSF pattern, ranging from 0.5 to 6 Å, with average values of 1.1 and 0.9 Å, respectively.

**Fig. 5:**
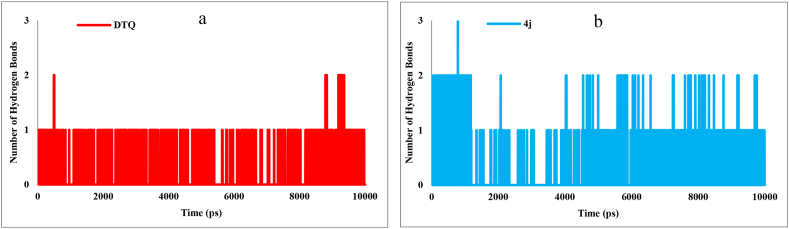
The number of hydrogen bonding interactions of (a) DTQ and (b) **4j** within the active site of CDK2 during 100 ns simulation.

To further evaluate the stability of the protein-ligand complex, hydrogen bonding interactions were analyzed throughout the MD simulation. The calculation and analysis of hydrogen bonding interactions were carried out by VMD (Visual Molecular Dynamics), version 1.9.3 [38], and the H-bond module in GROMACS. The default hydrogen bonding criteria were set as d ≤ 3.5 Å and θ (X-H–A)≥20° for the calculations. The number of hydrogen bonds formed by **4j** and DTQ within the active site of CDK2 during the simulation is depicted in Fig. 5. The findings indicated that he DTQ exhibited three hydrogen bonds throughout the simulation (Fig. 5a), whereas the **4j** formed four hydrogen bonds (Fig. 5b). Additionally, the occupancy percentages, which indicate the stability and the lifetime of each hydrogen bond are presented in Table 3. According to these results, **4j** established the most stable hydrogen bonds with ASP145 and LYS33, and DTQ formed hydrogen bonds with LEU83 and ASP145. These are consistent with the docking result, suggesting that compound **4j** maintains the same binding mode with the CDK2 active site during simulation.

**Table 3:** Hydrogen bond analysis of compound **4j** and DTQ within the active site of CDK2.

Donor	Acceptor (Occupancy)	Donor	Acceptor (Occupancy)
**4j**	ASP145 (8.39 %)	LEU83	DTQ (15.25 %)
LYS33	**4j** (4.36 %)	DTQ	ASP145 (2.66 %)
LYS33 **4j** LYS89	**4j** (9.73 %) TYR15 (1.11 %) **4j (**1.90 %)		

The molecular mechanics Poisson Boltzmann surface area (MMPBSA) method was used to assess the binding free energy of compound **4j** and the native ligand DTQ. Decomposition energy analysis per residue was calculated to determine the contribution of each residue to the binding energy of the ligand in the CDK2 complex. The binding energy terms and their contribution to the total binding energy of **4j** and DTQ at the active site of the CDK2 are listed in Table 4. Compound **4j** exhibited a binding energy (−87.14 ± 3.01 kJ/mol) comparable to DTQ (−81.46 ± 2.72 kJ/mol). The favorable energy term for **4j** binding energy was to be driven by the van der Waals (VDW) energy rather than the electrostatic energy. This justifies the low number of hydrogen bonds and their low occupancies obtained in the hydrogen bonding analysis. The hydrogen bonding represented in the electrostatic energy term was more prominent for compound **4j** (−39.255 ± 2.317 kJ/mol) than that observed for DTQ (−23.630 ± 2.135 kJ/mol). This finding corroborates our earlier observations regarding the higher number of hydrogen bonds formed by compound **4j** than DTQ. Moreover, **4j** showed the VDW energy value of −169.51 ± 1.93 kJ/mol, which was more negative than DTQ (−138.15 ± 1.88). This is in line with our finding in docking studies in which **4j** mainly established hydrophobic interactions with the hydrophobic residues of the active site through trimethoxyphenyl, pyranon, and ethyl moieties.

**Table 4:** Binding affinities and individual energy components resulting from MM-PBSA analysis of **4j** and DTQ complexes.

Compound	ΔEvdw (kJ/mol)	ΔEelec (kJ/mol)	ΔGpolar (kJ/mol)	ΔGnonpolar (kJ/mol)	ΔGbind (kJ/mol)
**4j**	−169.51 ± 1.93	−39.25 ± 2.32	142.92 ± 4.79	−21.24 ± 0.16	−87.14 ± 3.01
DTQ	−138.15 ± 1.88	−23.63 ± 2.13	96.15 ± 2.18	−15.73 ± 0.23	−81.46 ± 2.72

**Fig. 6:**
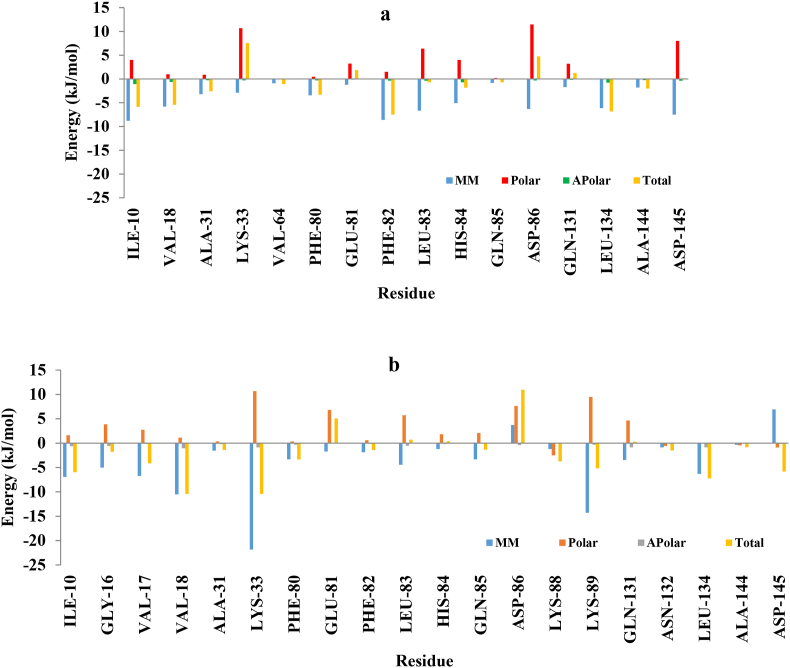
The per-residue decomposition energy analysis graph for the (a) DTQ-CDK2 and (b) **4j**-CDK2 complexes.

The per-residue decomposition energy analysis graph for the DTQ-CDK2 and **4j**-CDK2 complexes is depicted in Fig. 6(**a** and **b**). Accordingly, ILE10, VAL17, VAL18, ALA31, LYS33, PHE80, PHE82, GLN131, ASN132, LUE134, ALA144, and ASP145 of CDK2 played pivotal roles in the stabilization of **4j** bound to the receptor. This is consistent with the results of molecular docking and hydrogen bond analysis.

### 3.6 *In-silico* prediction of drug-likeness and pharmacokinetic properties

All the designed derivatives, except **4g**, fulfill the drug-likeness rules, including Lipinski [39], Ghose [40], Veber [41], Egan [42], and Muegge [43]. Compound **4g** failed to comply with Veber and Egan rules [44] (Table 5).

**Table 5:** The drug-likeness and pharmacokinetic properties predicted results for **4a**-**j**.

Drug-likeness						Pharmacokinetics					
Code	Lipinski	Ghose	Veber	Egan	Muegge	GI absorption	BBB Permeation^a^	P-gp Substrate	P-gp inhibition	Caco-2^b^	MDCK^c^
**4a**	Yes	Yes	Yes	Yes	Yes	High	0.559	No	Non	18.69	15.13
**4b**	Yes	Yes	Yes	Yes	Yes	High	0.348	No	Non	13.62	5.89
**4c**	Yes	Yes	Yes	Yes	Yes	High	0.093	No	Non	21.36	1.97
**4d**	Yes	Yes	Yes	Yes	Yes	High	0.846	No	Non	19.83	7.97
**4e**	Yes	Yes	Yes	Yes	Yes	High	0.020	No	Non	11.36	10.05
**4f**	Yes	Yes	Yes	Yes	Yes	High	0.162	No	Non	21.12	0.29
**4g**	Yes	Yes	No	No	Yes	Low	0.009	Yes	Inhibitor	5.89	15.79
**4h**	Yes	Yes	Yes	Yes	Yes	High	0.014	No	Inhibitor	10.72	1.42
**4i**	Yes	Yes	Yes	Yes	Yes	High	0.120	No	Non	21.55	7.97
**4j**	Yes	Yes	Yes	Yes	Yes	High	0.143	No	Inhibitor	22.63	2.59

a *In vivo* blood-brain barrier penetration (C.brain/C.blood); Low: <0.1, Moderate: 0.1–2, High: >2.

b *In vitro* Caco-2 (Human colorectal carcinoma) cell permeability (nm/s); <4: low, 4–70: moderate, >70: high.

c *In vitro* Madin-Darby Canine Kidney (MDCK) cell permeability (nm/s); <25: low, 25–500: moderate, >500: high.

The estimated pharmacokinetic profiles of **4a**-**j** are listed in Table 5. Moreover, all compounds were predicted to have high GI absorption, except for **4g**, which showed low GI absorption, indicating that other compounds are well orally absorbed. All the derivatives presented low to moderate blood-brain barrier (BBB) permeation; consequently, they are less likely to cause neurotoxicity (**4g**, **4h**, and **4e** have the lowest BBB permeation). All the derivatives showed medium permeability in human colorectal carcinoma cells (Caco-2) and low permeability in Madin-Darby Canine Kidney cells (MDCK). All derivatives, except **4g**, are not P-glycoprotein (P-gp) substrates [45]. P-gp has been identified as the most important efflux transporter responsible for multidrug resistance (MDR) of cancer cells to chemotherapeutic agents pumping anti-cancer drugs outside the cell. Therefore, the anti-cancer potency of the compounds might not be affected by P-gp. Compounds **4g**, **4h**, and **4j** were predicted to have an inhibitory effect on the P-gp pump. Hence, co-administration of these cytotoxic agents with common anti-cancer drugs might reverse MDR.

### 3.7 DFT approach

**Fig. 7:**
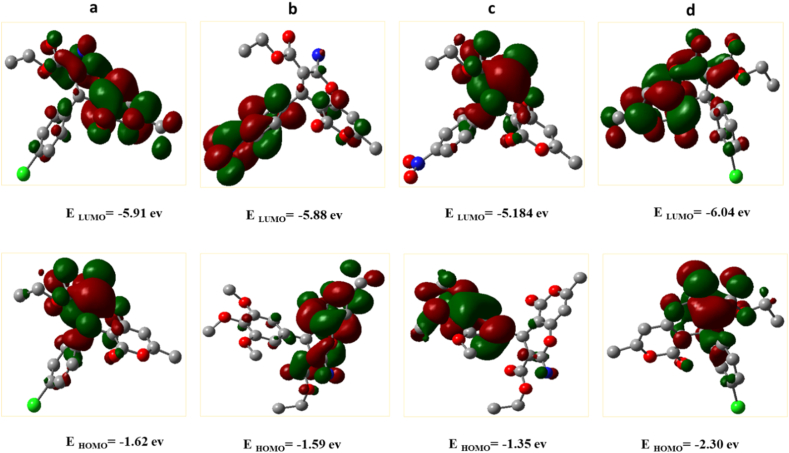
DFT calculated HOMO, LUMO, and their energies for (a) **4i**, (b) **4j**, (c) **4g**, and (d) **4d** at the B3LYP/6–311++G (d,p) level of theory.

Molecular orbitals such as HOMO and LUMO, as well as the energy gap between them (ΔEgap), determine the chemical reaction site and molecular kinetic stability. HOMO energy indicates the capacity of the molecule to donate electrons, and LUMO energy indicates the capacity of the molecule to accept electrons. HOMO and LUMO energies using B3LYP/6–311++G (d,p) were measured and are presented in Fig. 7. The energy gaps between HOMO and LUMO were obtained at 4.293, 4.293, 3.834, and 3.744 eV for **4i**, **4j**, **4g**, and **4d**, respectively. A high energy gap indicates that the ligand is more stable, while a low energy gap indicates that the ligand is more reactive. In many cases, the energy gap confirms the biological behavior of a molecule. The energy gaps for **4i**, **4j**, and **4g** derivatives were lower than that of derivative **4d**, which indicated more stability of these derivatives. As can be seen, the LUMO of **4i** derivative is concentrated on the whole molecule, and the HOMO of this compound is distributed in most parts of the molecule except the pyranon ring (Fig. 7a). For compound **4j**, LUMO is located on the entire molecule except the ethyl carboxylate group and HOMO is on the dihydropyranopyran core, NH_2_, and carboxylate moiety (Fig. 7b). For compound **4g**, LUMO is placed on the entire compound except the 4-nitrophenyl ring, while HOMO is placed only on this ring (Fig. 7c). In the case of **4d**, HOMO and LUMO are distributed throughout the molecule (Fig. 7d).

**Fig. 8:**
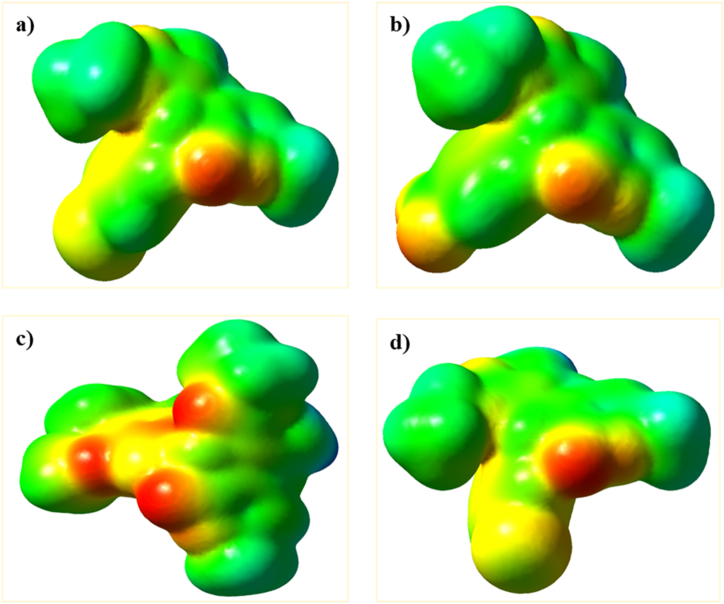
ESP calculated for (a) **4i**, (b) **4g**, (c) **4j,** and (d) **4d** at B3LYP/6–311++G (d,p) level of theory.

Electrostatic surface potential (ESP) describes the electron density around a molecule and is a useful indicator for determining nucleophilic and electrophilic sites. The diagrams for the compounds **4i**, **4j**, **4g**, and **4d** are plotted in Fig. 8(a-d). The negative regions represent the electrophilic sites (red color), while the positive regions indicate the nucleophilic centers (blue color).

Theoretical chemistry can help to estimate the reactivity or stability of chemical species. Quantum chemical descriptors predict the chemical reactivity of molecules, the analysis of reactions, and the location of reactions in molecules. Thermochemical parameters such as total energy (E_tot_), enthalpy energy (H), Gibbs energy (G), and entropy energy (S) were measured based on the B3LYP/6–311++G (d,p) level of theory. Moreover, hardness (η), softness (σ), and electron affinity (A) were determined using HOMO and LUMO energies. The values of chemical reactivity indices for derivatives **4i**, **4g**, **4j**, and **4d** are presented in Table 6. According to the results, the energy values of **4i**, **4j**, and **4g** derivatives are more than **4d**, so these compounds are more stable. Additionally, the energy value of the **4i** derivative shows a higher stability of this molecule than other compounds. It is noticed that molecules with a larger energy gap are harder and have higher kinetic stability because of resistance to electron cloud deformation. Considering that **4i**, **4g**, and **4j** derivatives have a larger energy gap, the hardness parameter of these compounds is expected to be higher than that of compound **4d**. The value of electron affinity for compound **4d** is calculated to be higher than other compounds, indicating the greater tendency of this compound to accept electrons, which is consistent with other obtained results.

**Table 6:** The chemical reactivity indices of **4i**, **4g**, **4j**, and **4d** at B3LYP/6–311++G (d,p) level of theory.

Entry	E_tot_^a^	H^a^	G^a^	S^b^	ɳ^c^	σ^d^	A^c^
**4i**	−1577.922	−1577.921	−1577.997	159.629	2.146	0.232	1.62
**4g**	−1577.920	−1577.919	−1577.996	161.869	2.146	0.232	1.593
**4j**	−1462.083	−1462.082	−1462.174	194.183	1.917	0.260	1.35
**4d**	−1323.858	−1323.857	−1323.934	162.696	1.872	0.267	2.308

a In Hartree/particle.

b In cal/mol.K.

c In eV.

d In eV^−1^.

**Fig. 9:**
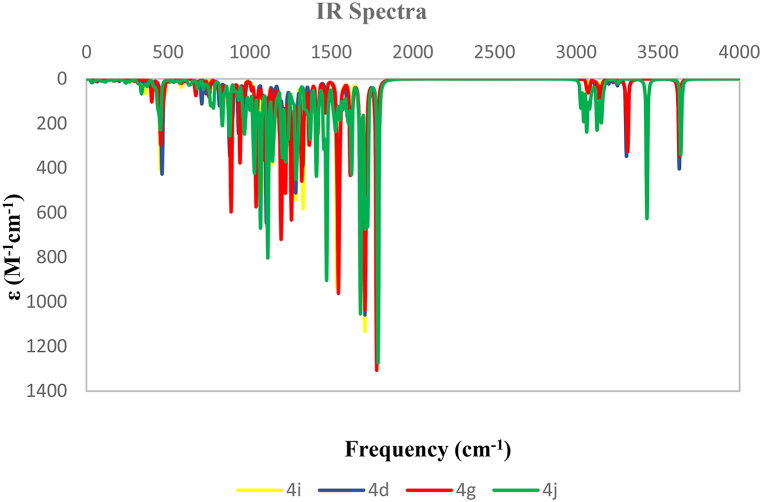
Calculated IR spectra of **4i** (yellow), **4d** (blue), **4g** (red), and **4j** (green) at B3LYP/6–311++G (d,p) level of theory. (For interpretation of the references to color in this figure legend, the reader is referred to the Web version of this article.)

The IR peaks were theoretically calculated at the B3LYP/6–311++G (d,p) level of theory for all derivatives, as shown in Fig. 9. By using the IR spectrum, aromatic and aliphatic C–H, C=O, and NH stretching vibrations can be seen clearly, which help to identify compounds’ structures. C=O stretching vibrations in the 1778.22, 1776.45, 1786.66, and 1779.31 cm^−1^ regions were observed for **4i**, **4g**, **4j**, and **4d** compounds, respectively. Furthermore, the C–H aromatic stretching vibrations were at ∼3000 cm^−1^ region, and aliphatic vibrations were visible around ∼2900. NH stretching vibrations were observed in the range 3300–3500 cm^−1^. The results indicated that theoretical and experimental IR spectra were matched.

## 4 Conclusion

A series of 2-amino-7-methyl-5-oxo-4-phenyl-4,5-dihydropyrano[4,3-*b*]pyran-3-carboxylate derivatives (**4a-j**) were synthesized and tested for their anti-proliferative and antioxidant activities. All the derivatives showed anti-proliferative effects against the cancer cell lines SW-480 and MCF-7. Compound **4j**, bearing a 3,4,5-trimethoxy phenyl at the C_4_ position, was among the most potent derivatives with IC_50_ values of 38.6 and 26.6 μM against SW-480 and MCF-7 cells, respectively. Moreover, **4j** proved to be a DPPH radical scavenger with an EC_50_ value of 580 μM. Molecular docking studies revealed that all the compounds could bind to the CCDK2 active site with good affinity. Excellent drug-likeness profiling and promising pharmacokinetic properties were predicted for almost all the dihydropyranopyran derivatives by *in-silico* studies. The findings imply that **4j** might be considered a potential lead molecule for additional research in the field of anti-cancer drug development.

## Funding statement

This work was supported by the 10.13039/501100013041Vice-Chancellor for Research, Shiraz University of Medical Sciences, Iran (project number: 98-01-36-20851).

## Data availability statement

Data will be made available on request.

## CRediT authorship contribution statement

**Sara Ranjbar:** Writing – review & editing, Writing – original draft, Supervision, Project administration, Methodology, Formal analysis, Conceptualization. **Paria Sadeghian:** Methodology, Investigation. **Sara Khademian:** Methodology, Data curation. **Mina Emami:** Investigation, Formal analysis. **Zahra Pakrouh Jahromi:** Visualization, Software, Formal analysis. **Seyedeh Habibeh Mirmajidi:** Methodology, Investigation. **Fateme Zare:** Resources, Methodology. **Manica Negahdaripour:** Writing – original draft, Validation, Software. **Younes Ghasemi:** Writing – review & editing, Validation, Funding acquisition. **Mehdi Khoshneviszadeh:** Writing – review & editing, Supervision, Project administration.

## Declaration of competing interest

The authors declare that they have no known competing financial interests or personal relationships that could have appeared to influence the work reported in this paper.
