# Supplementary material for: 5-Oxo-dihydropyranopyran derivatives as anti-proliferative agents; synthesis, biological evaluation, molecular docking, MD simulation, DFT, and in-silico pharmacokinetic studies
Source: Heliyon. 2024 Apr 23;10(9):e29850. doi: 10.1016/j.heliyon.2024.e29850 (PMC11066326; doi:10.1016/j.heliyon.2024.e29850)

*Ethyl 2-amino-7-methyl-5-oxo-4-phenyl-4,5-dihydropyrano[4,3-b]pyran-3-carboxylate (4a)*

**
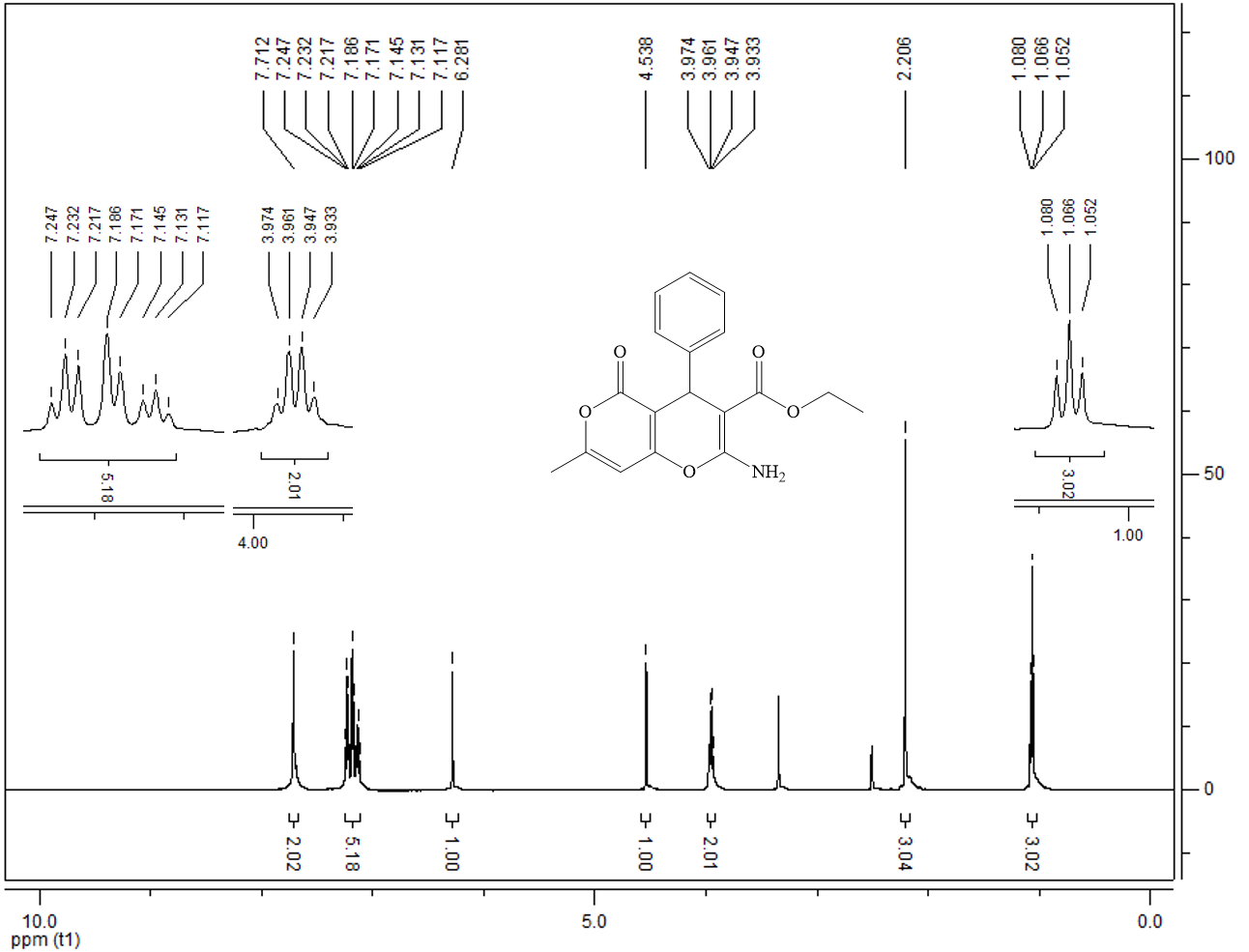
**


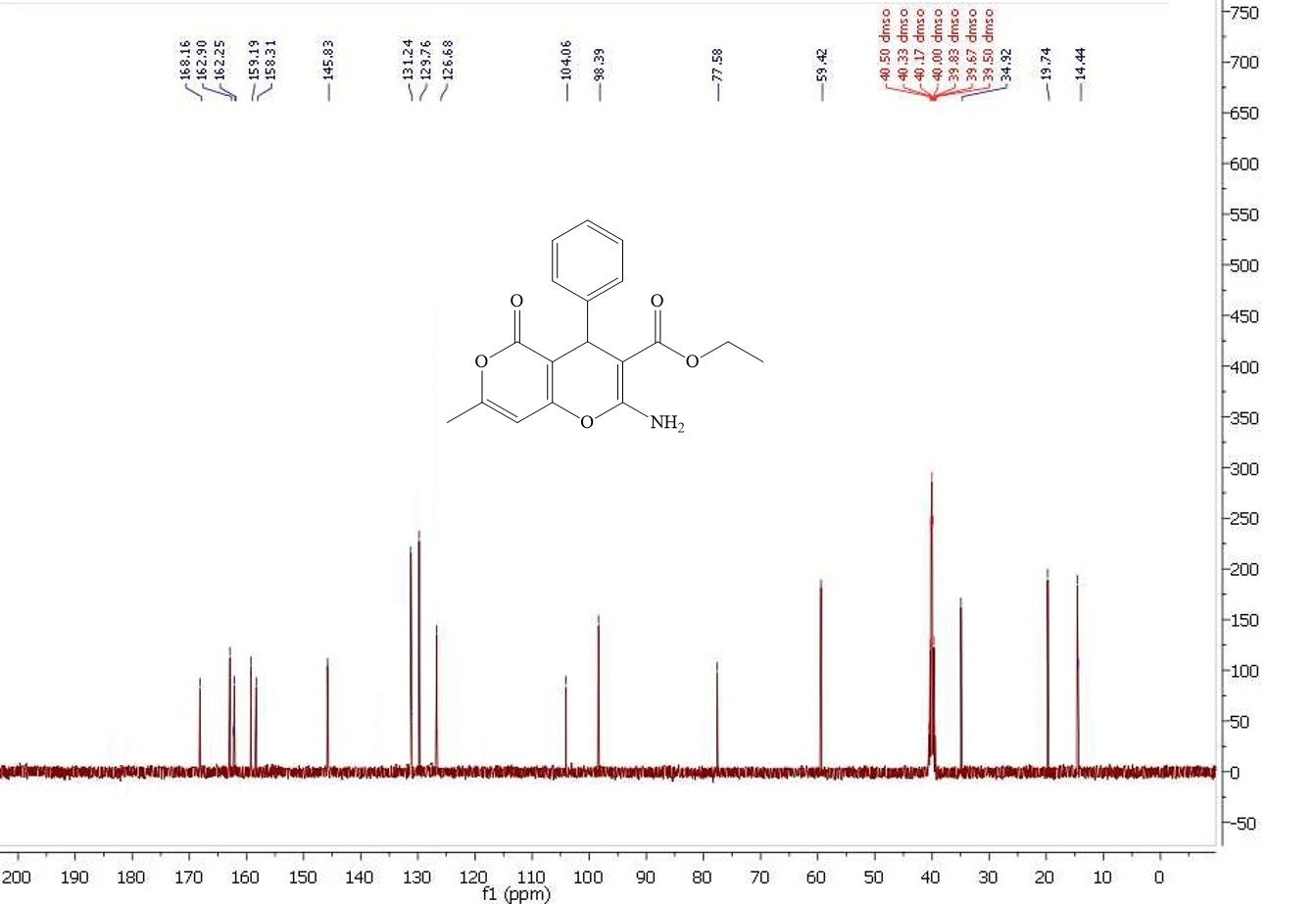


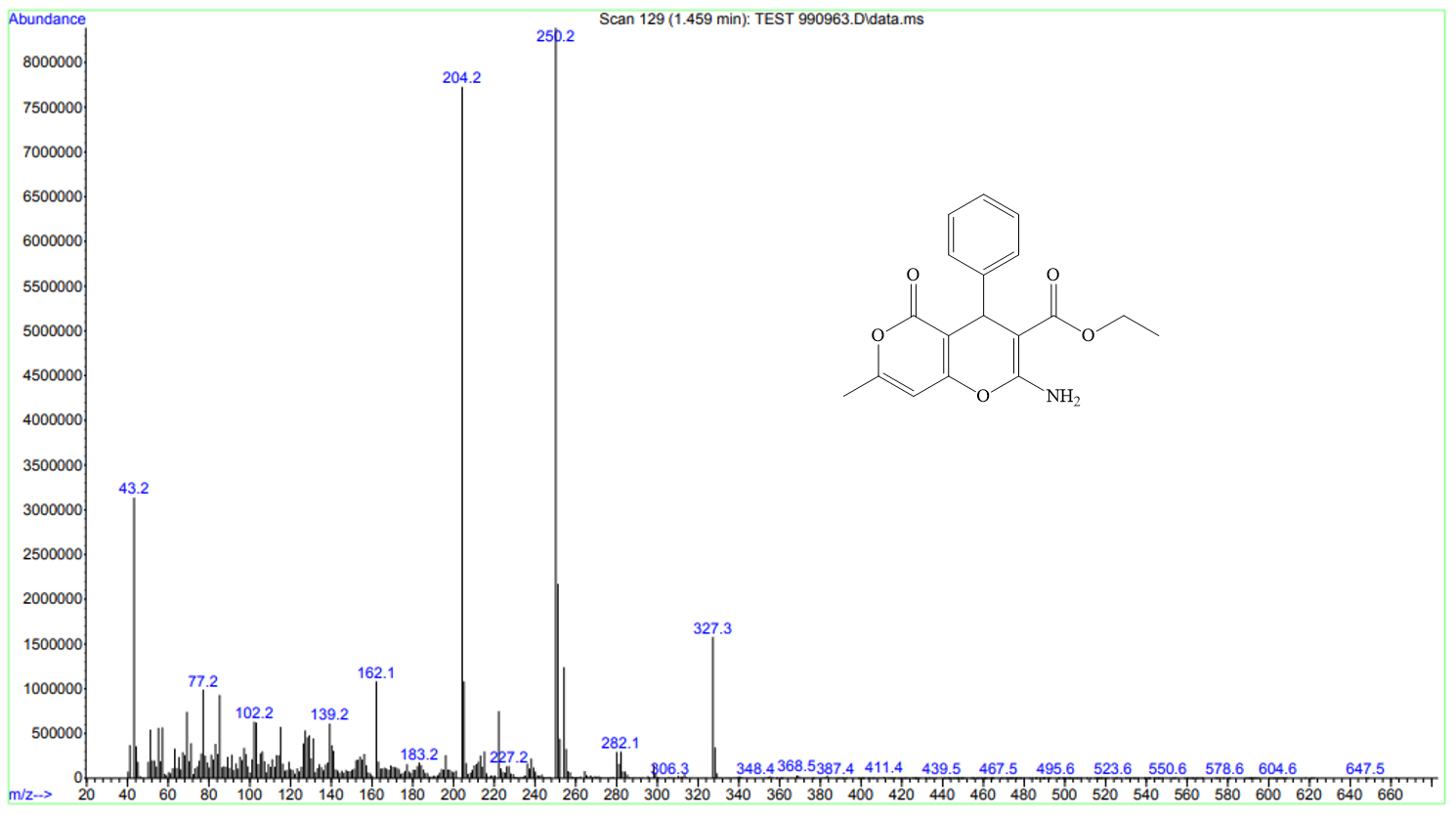


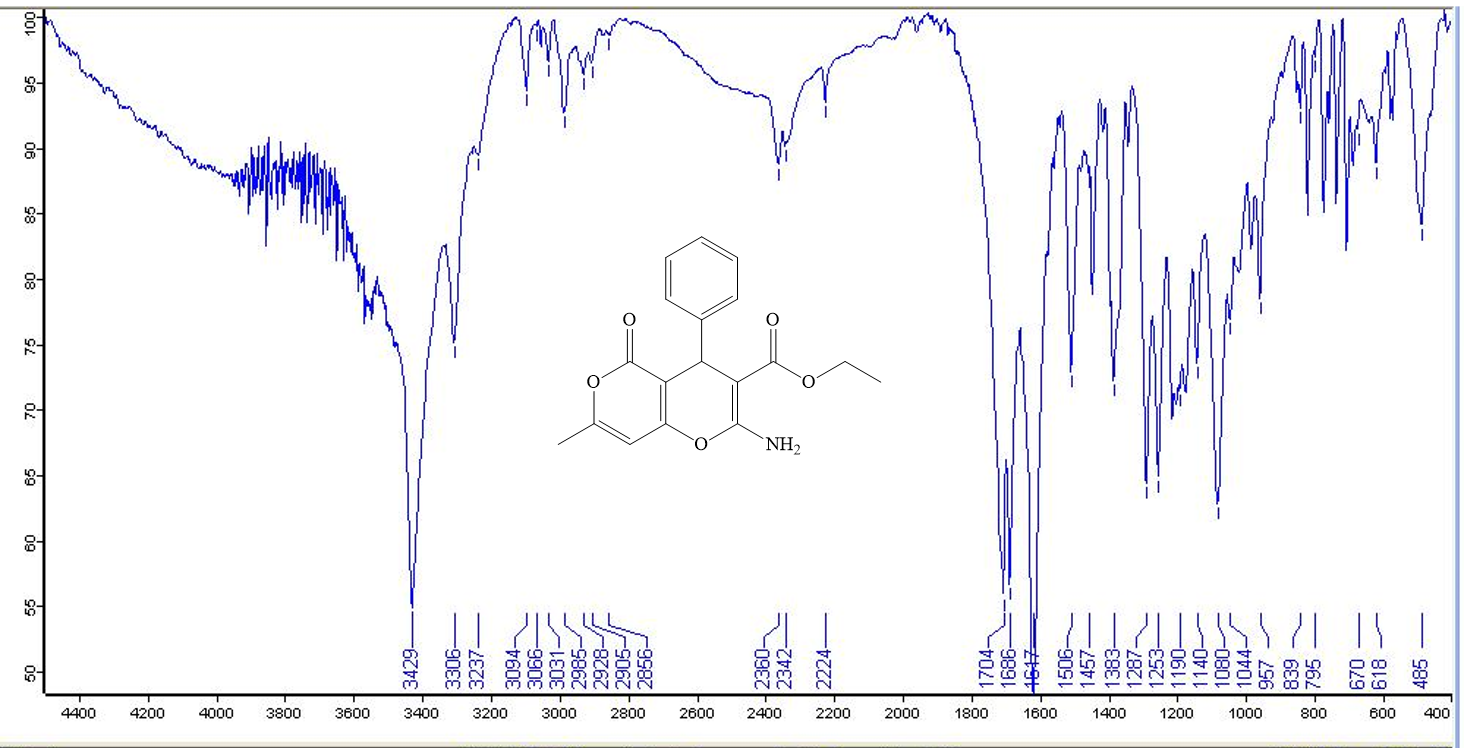


Ethyl 2-amino-4-(3-hydroxyphenyl)-7-methyl-5-oxo-4,5-dihydropyrano[4,3-b]pyran-3-carboxylate (4b)


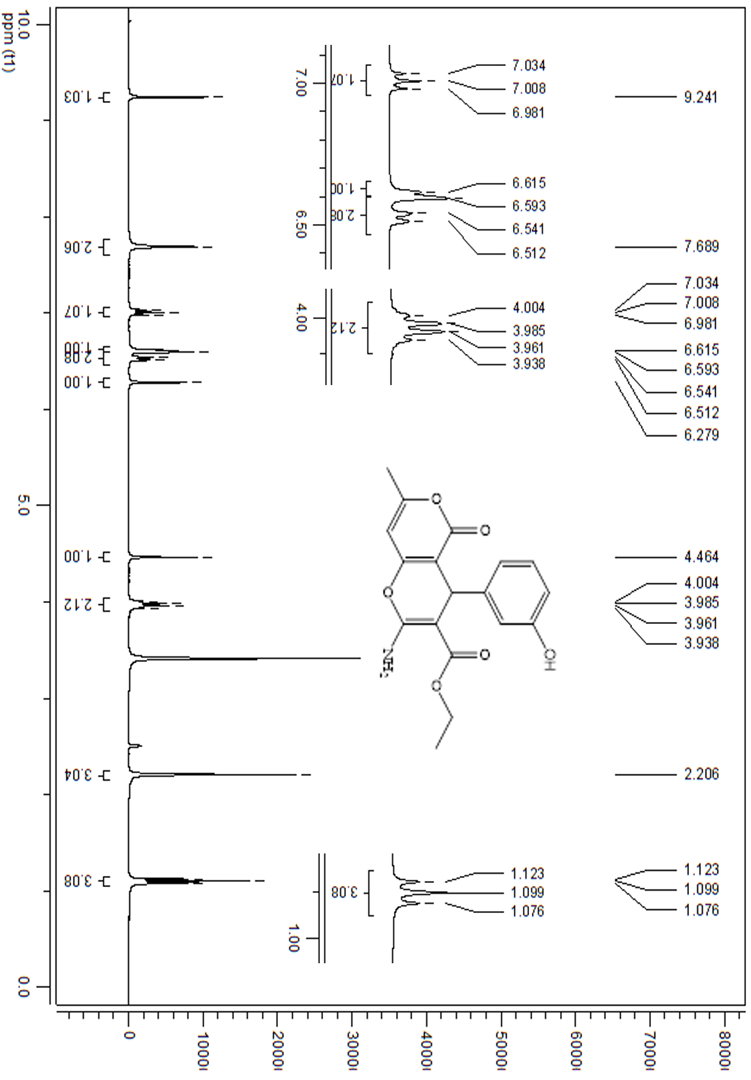


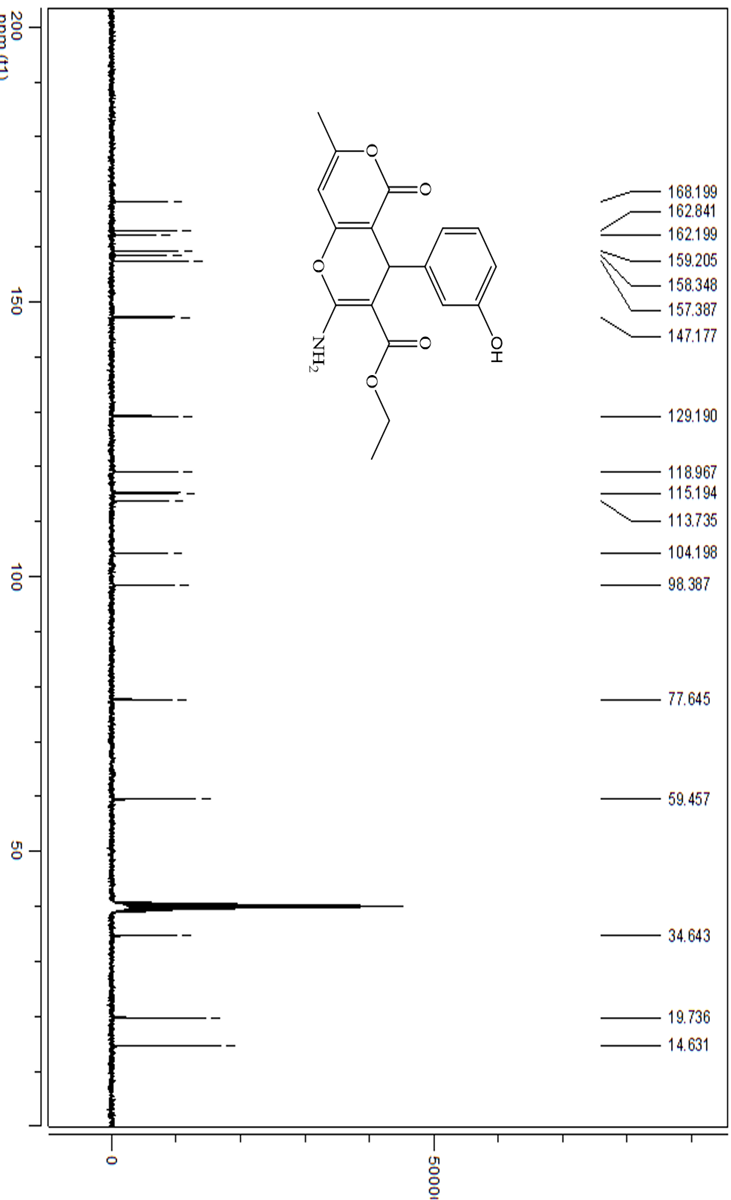


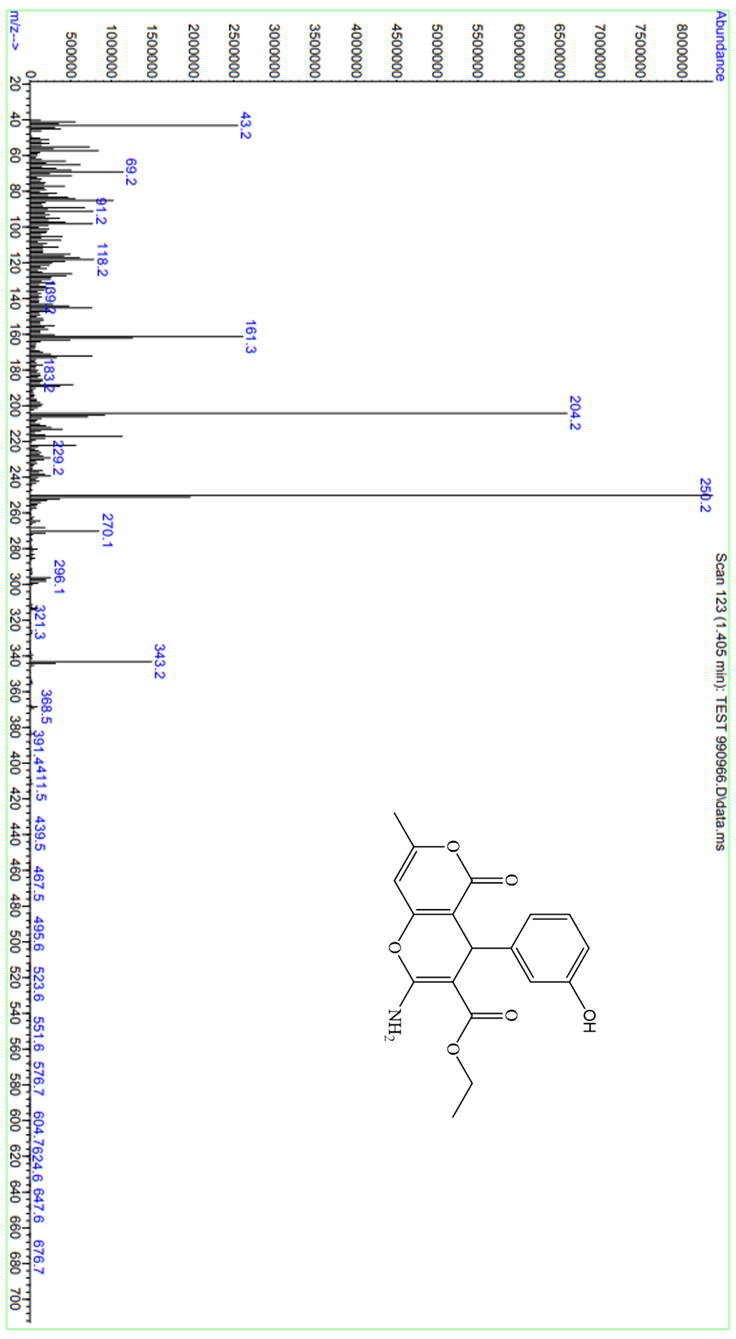


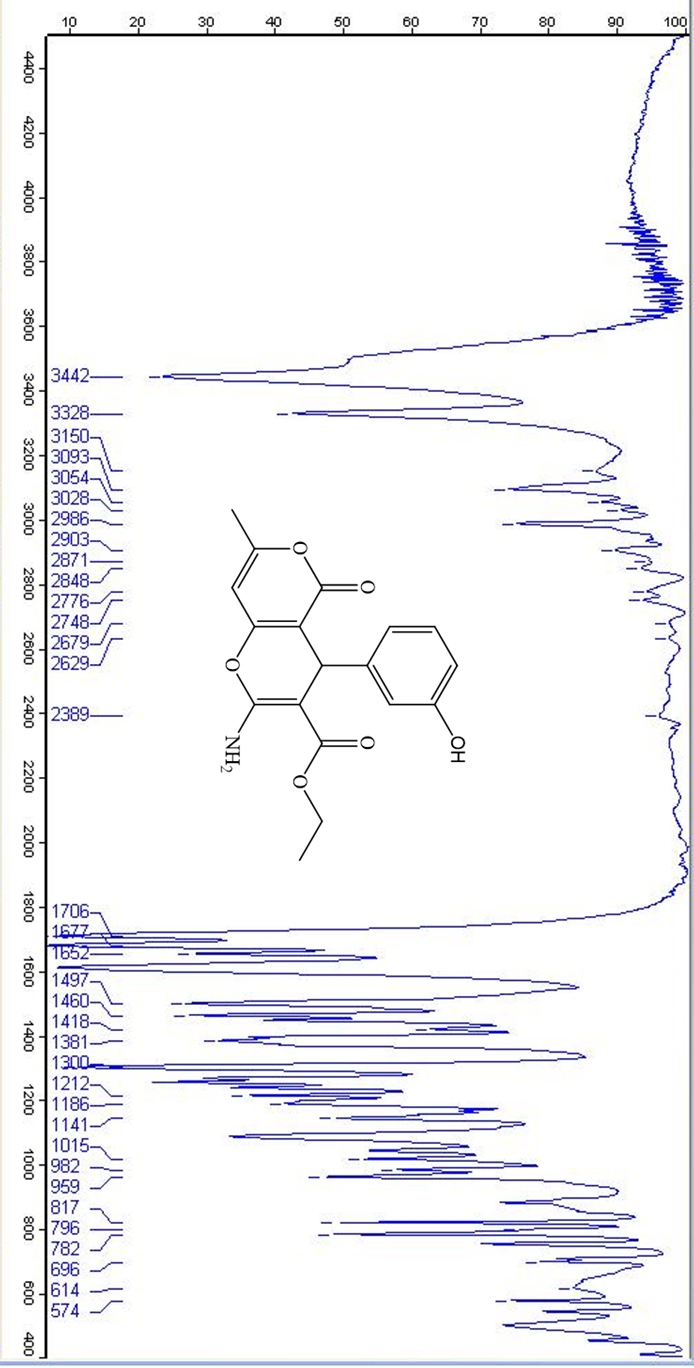


Ethyl 2-amino-4-(2,4-dichlorophenyl)-7-methyl-5-oxo-4,5-dihydropyrano[4,3-b] pyran-3-carboxylate(4c)


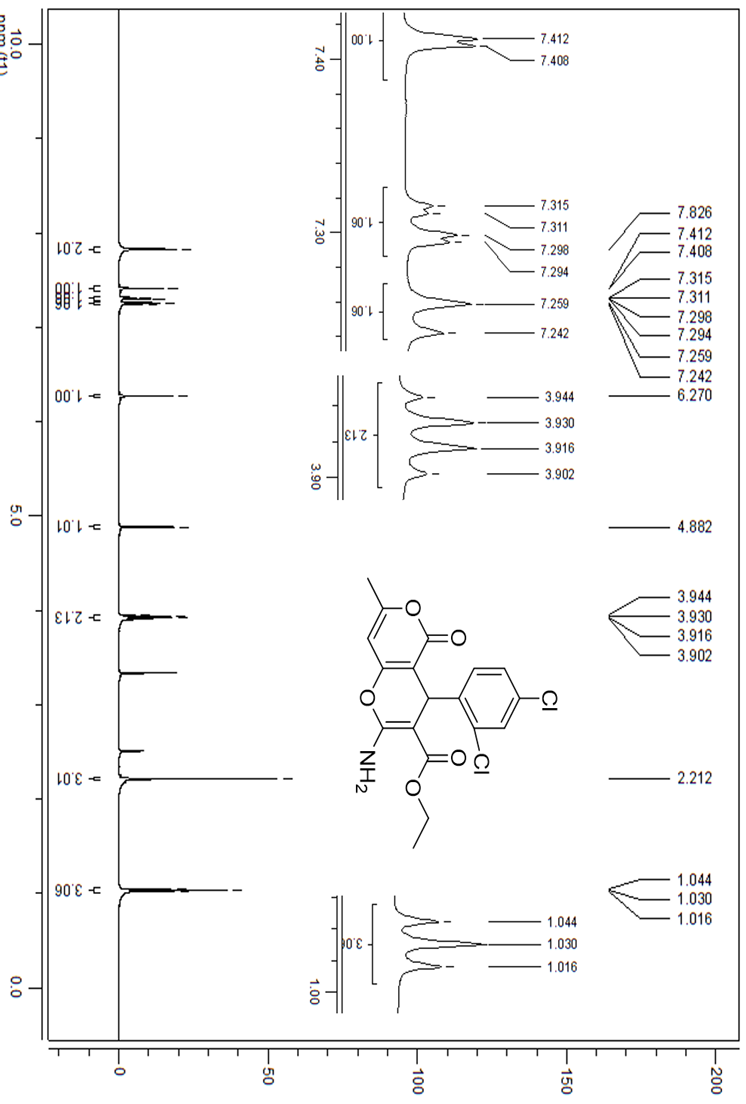


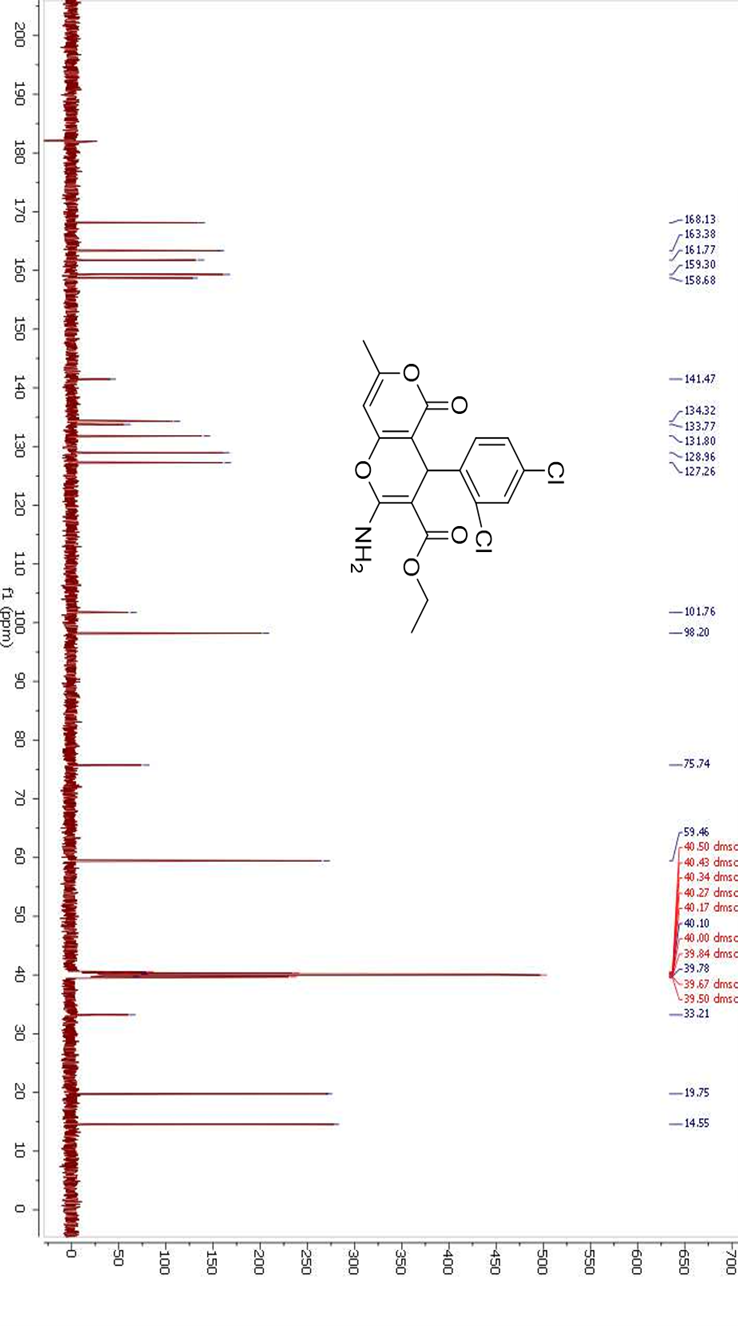


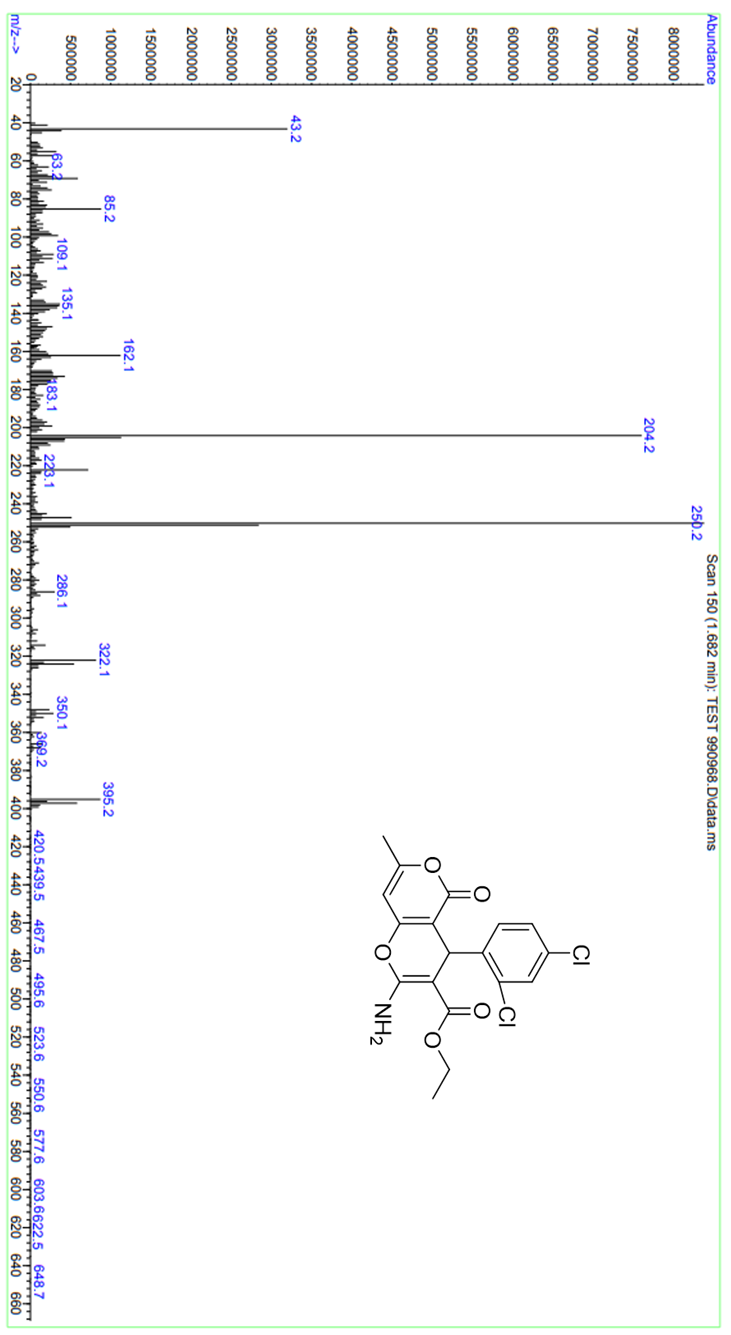


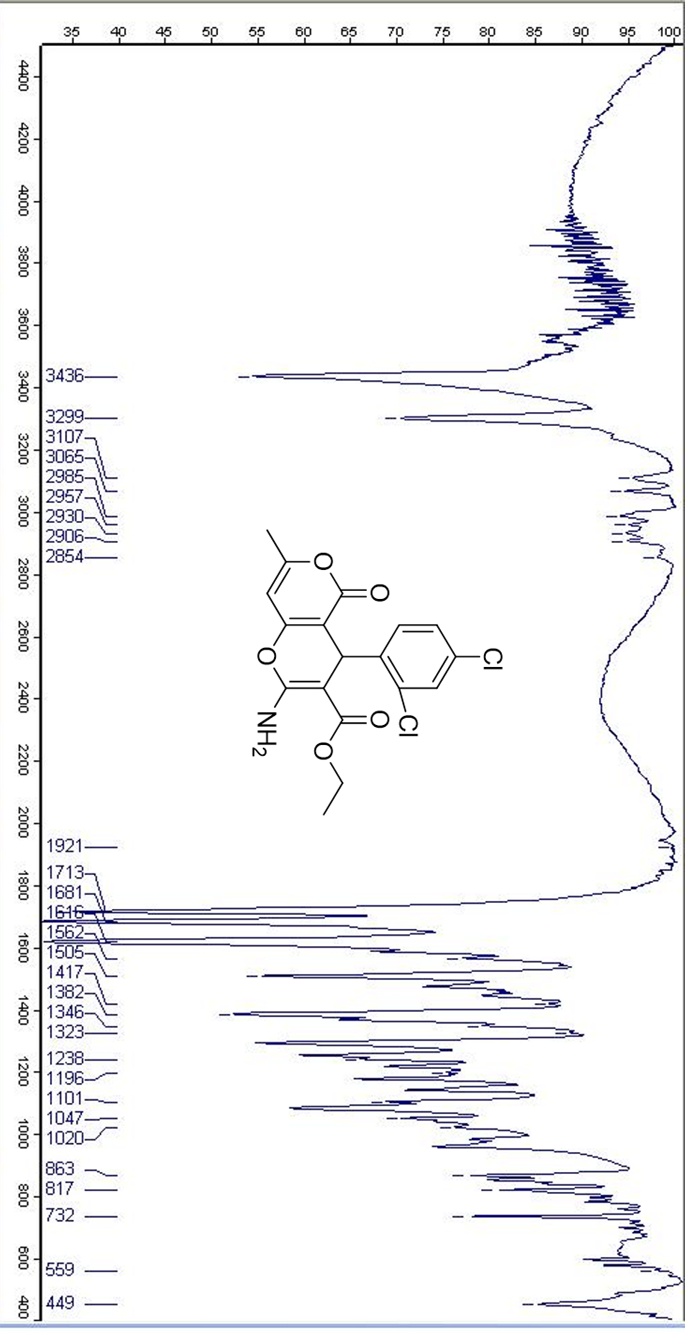


Ethyl 2-amino-4-(3-chlorophenyl)-7-methyl-5-oxo-4,5-dihydropyrano[4,3-b] pyran-3-carboxylate(4d)

**
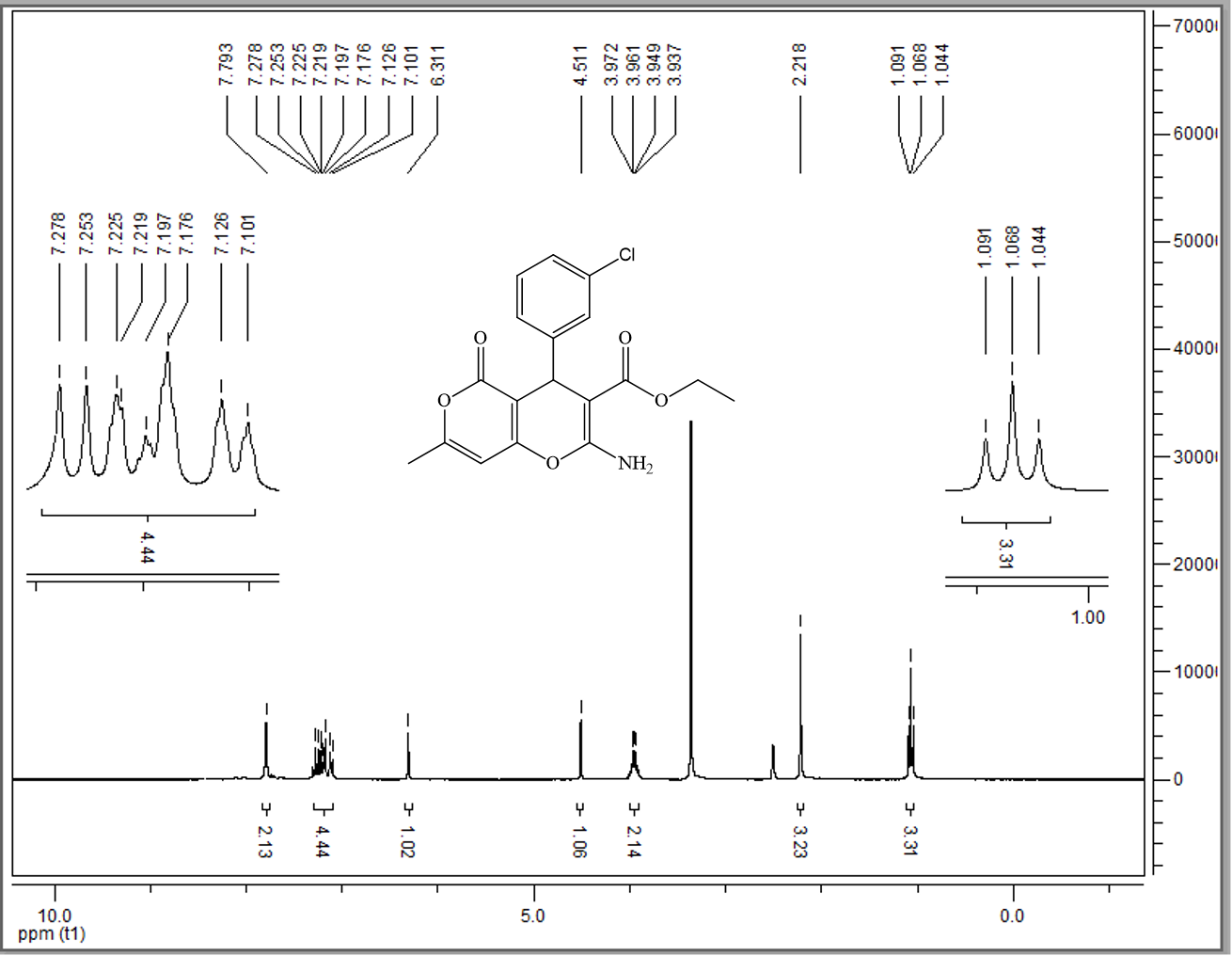
**


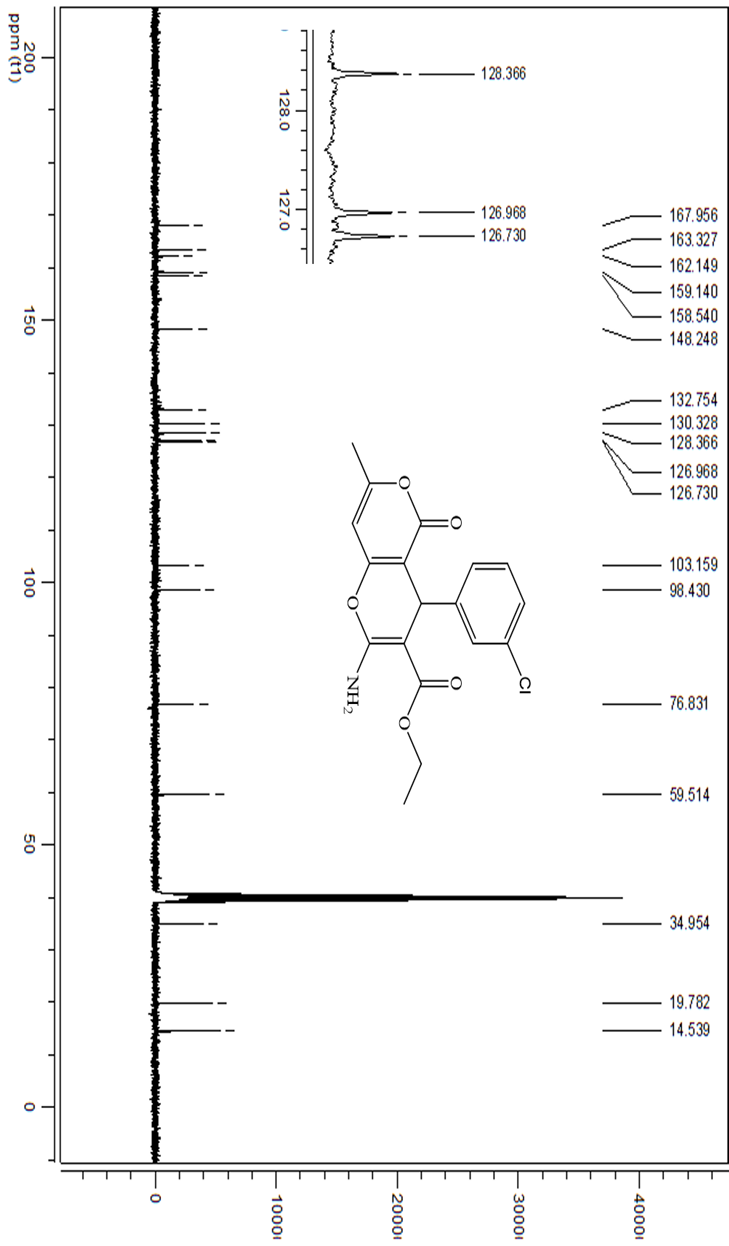


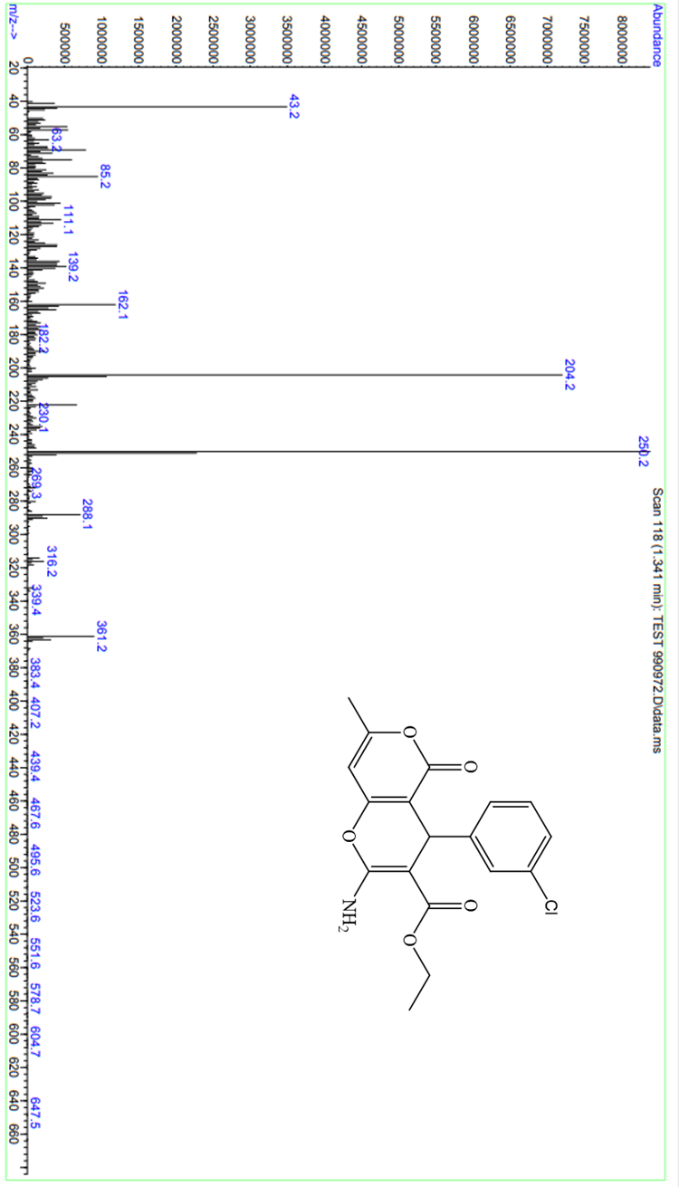


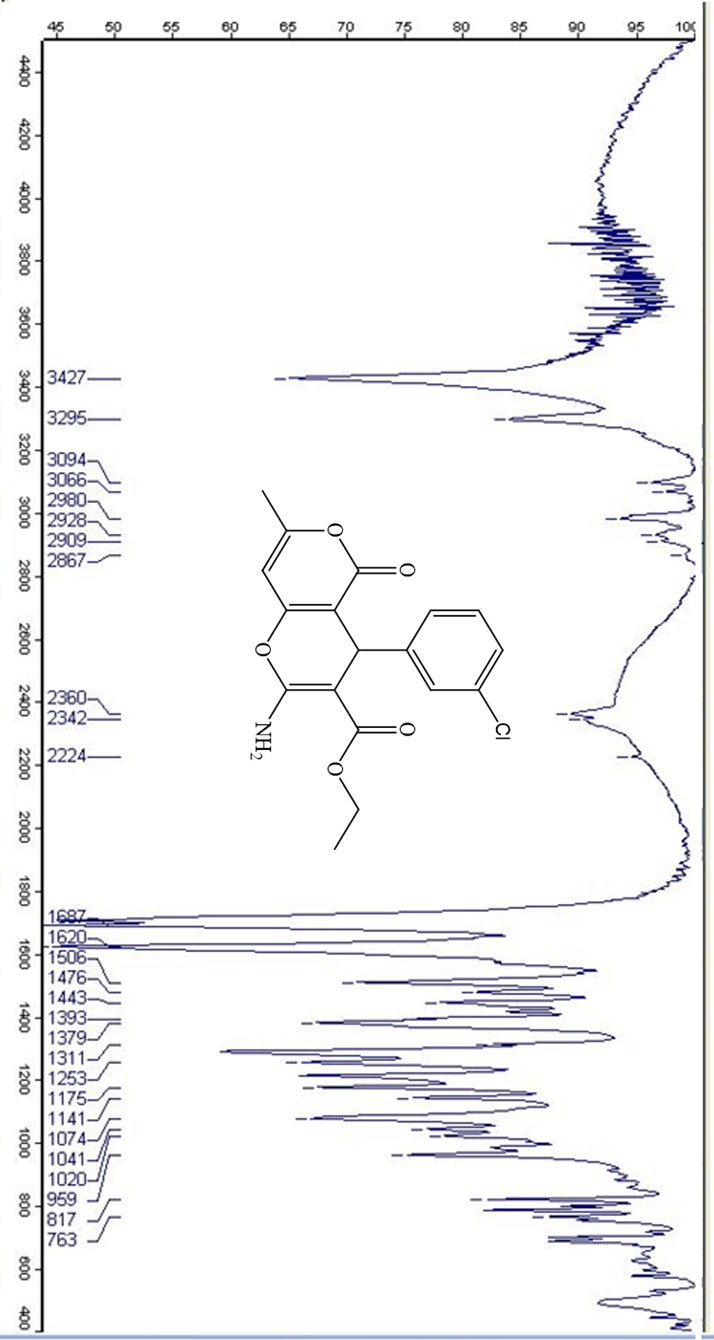


Ethyl 2-amino-4-(4-cyanophenyl)-7-methyl-5-oxo-4,5-dihydropyrano[4,3-b]pyran -3-carboxylate (4e)


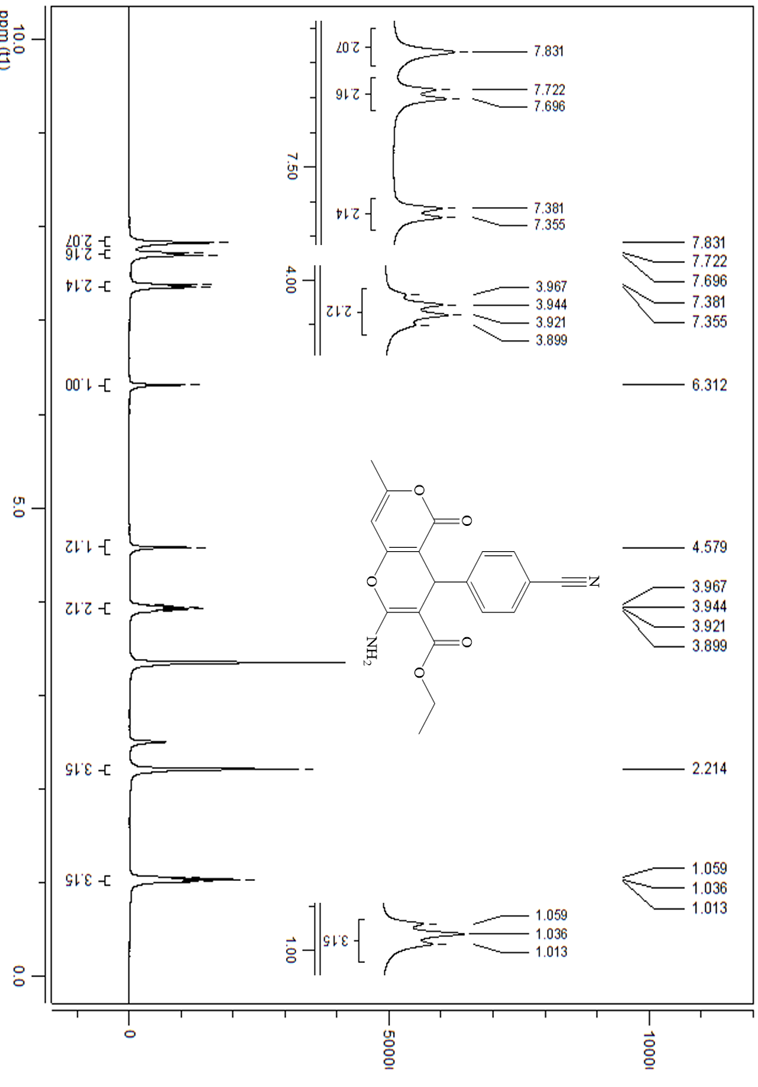


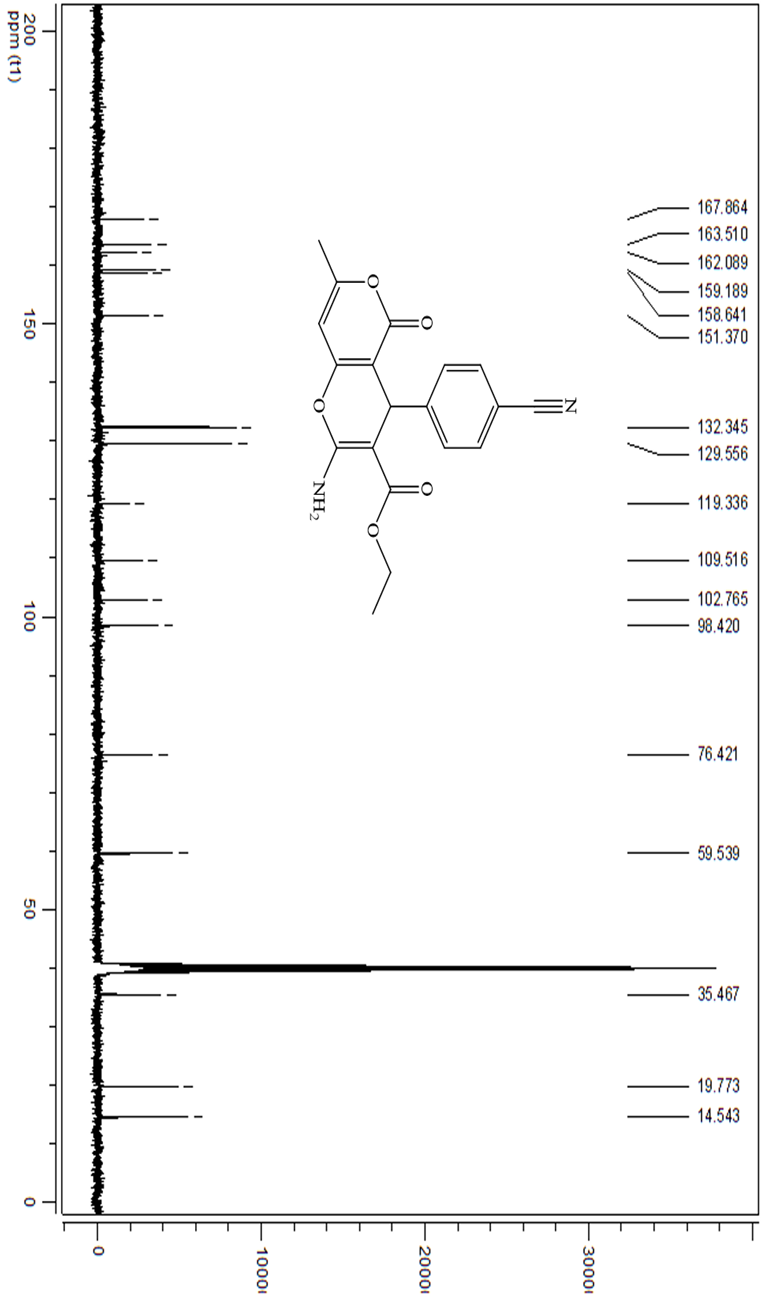


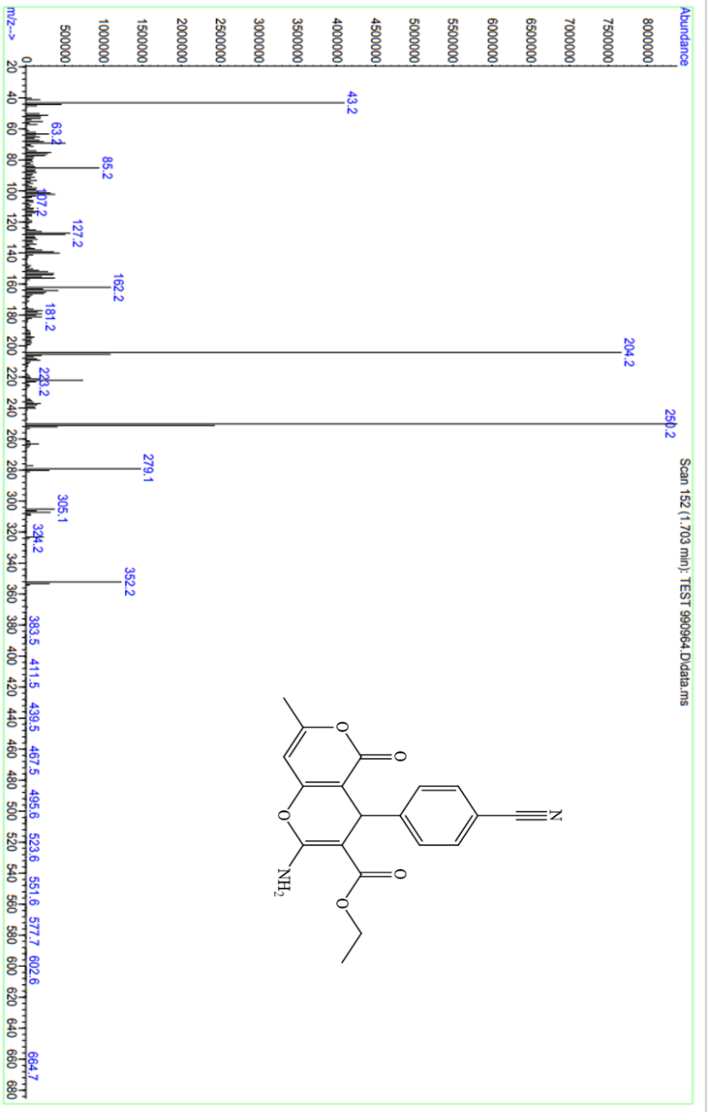


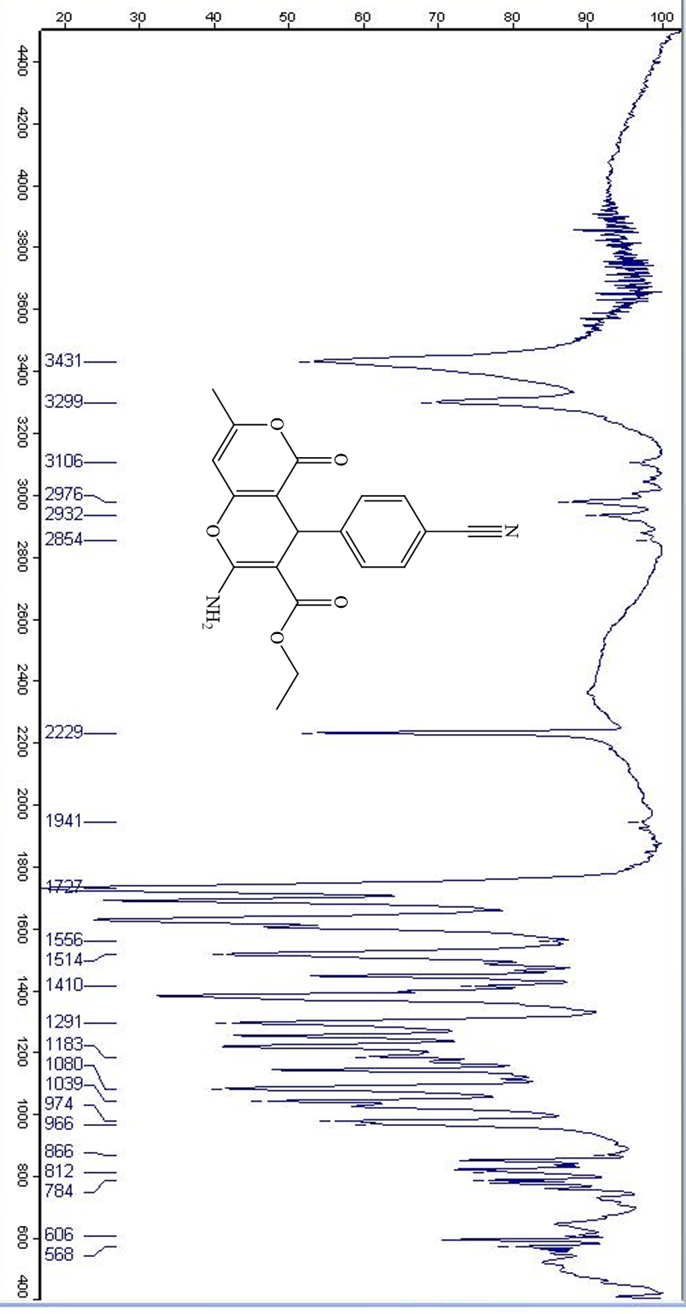


Ethyl 2-amino-4-(4-bromophenyl)-7-methyl-5-oxo-4,5-dihydropyrano[4,3-b] pyran -3-carboxylate(4f)


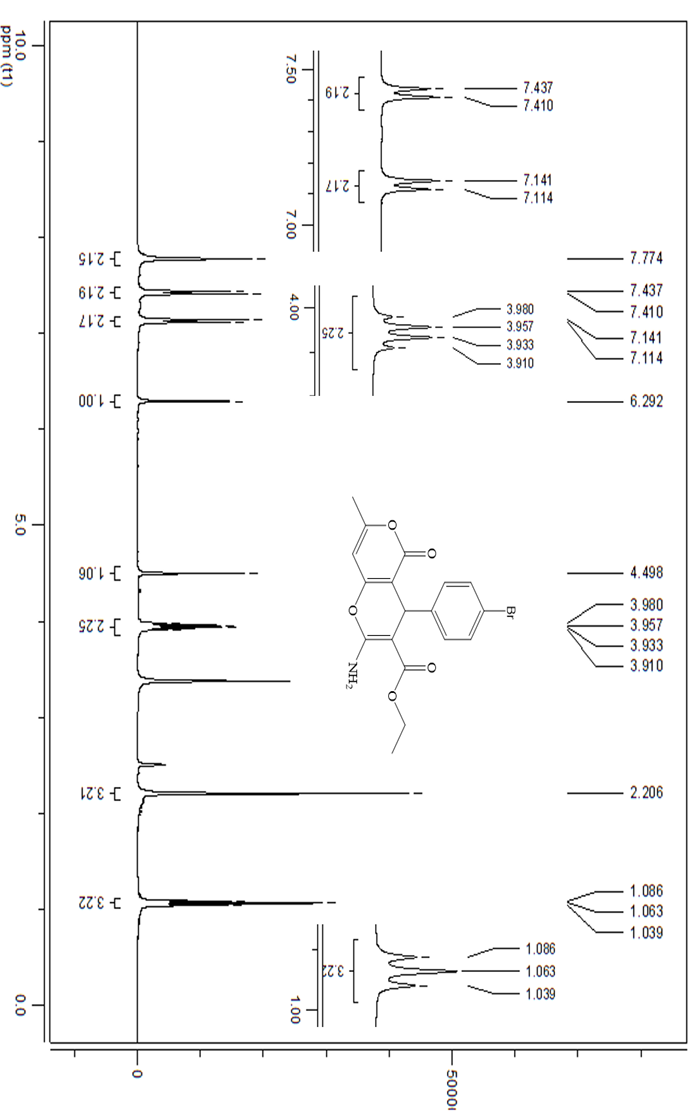


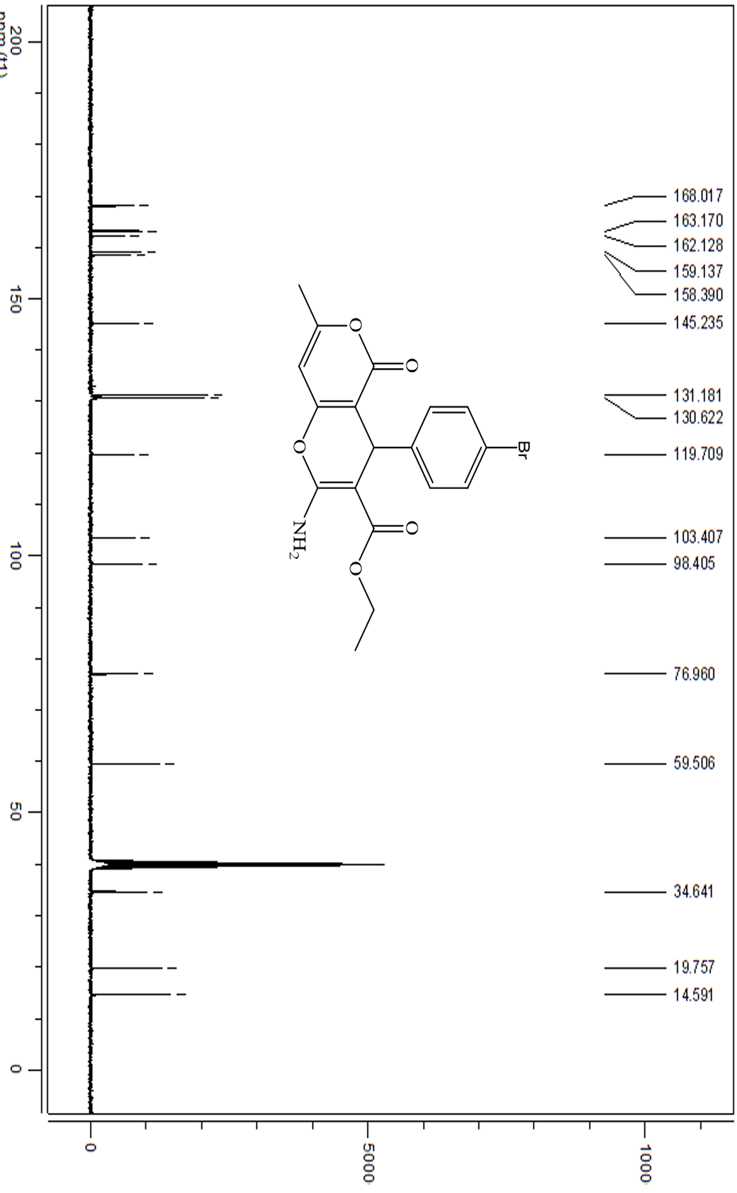


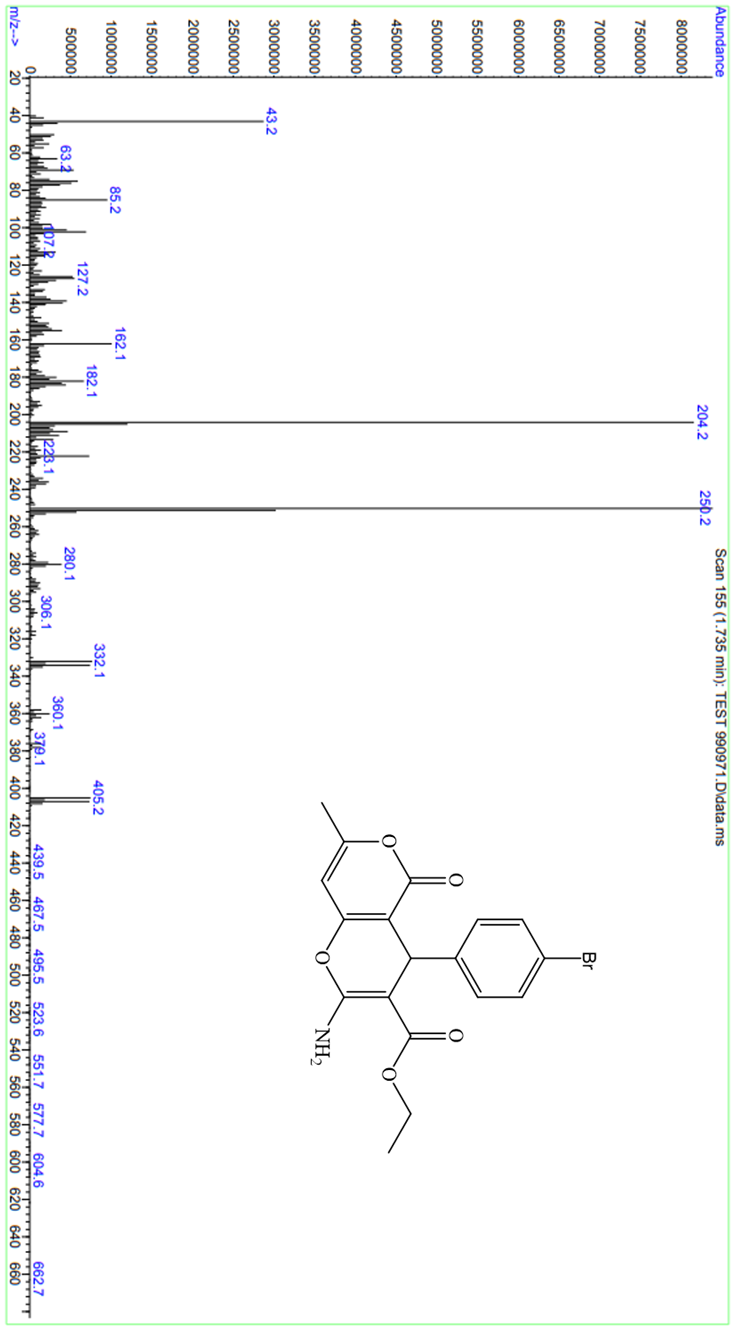


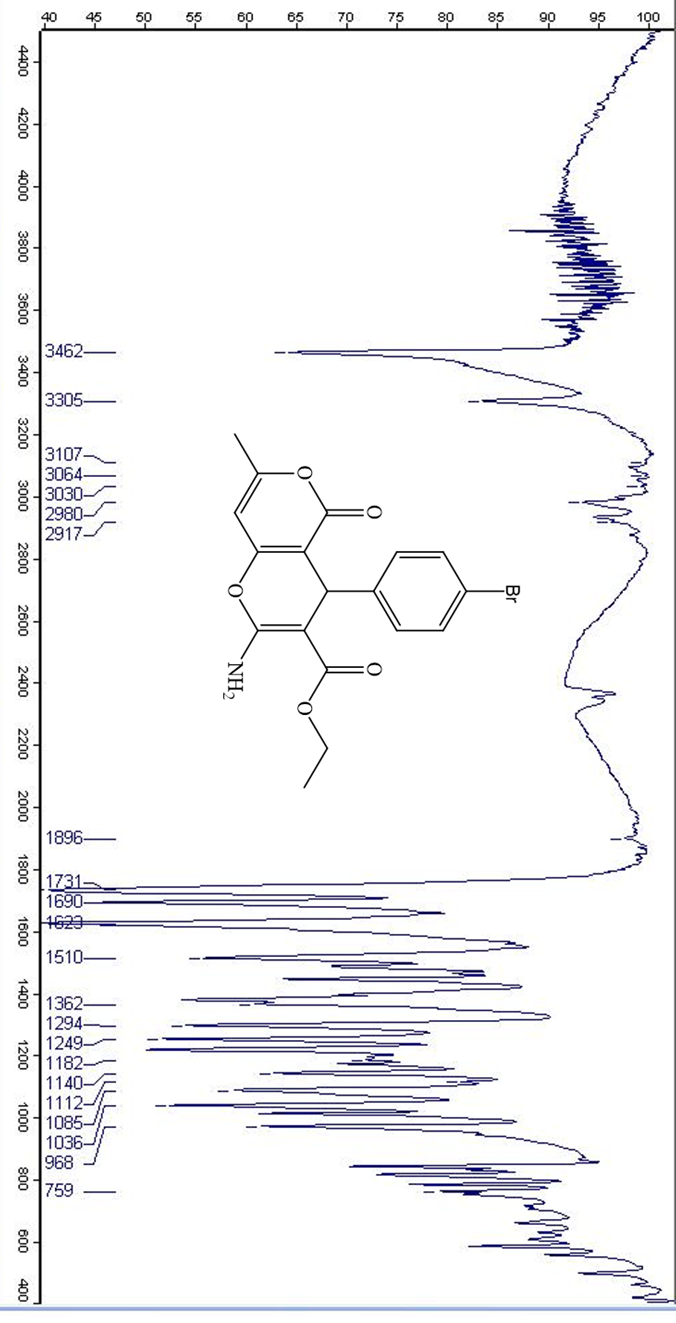


Ethyl 2-amino-7-methyl-4-(4-nitrophenyl)-5-oxo-4,5-dihydropyrano [4,3-b]pyran-3-carboxylate

(4g)


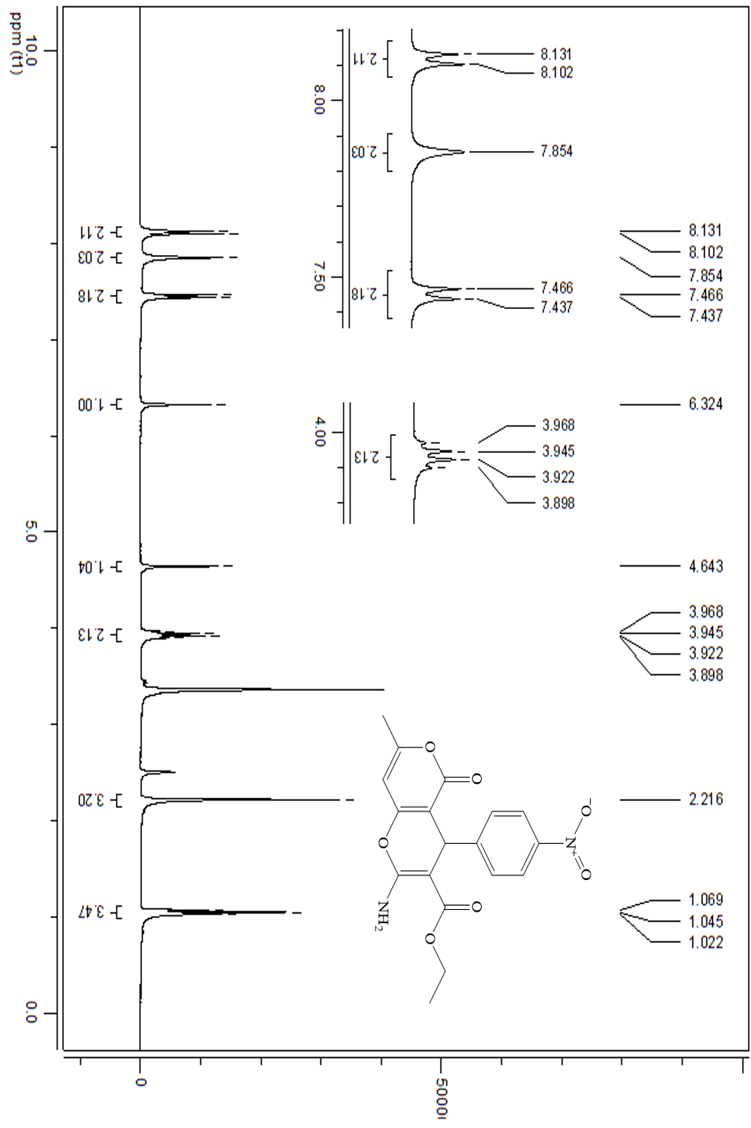


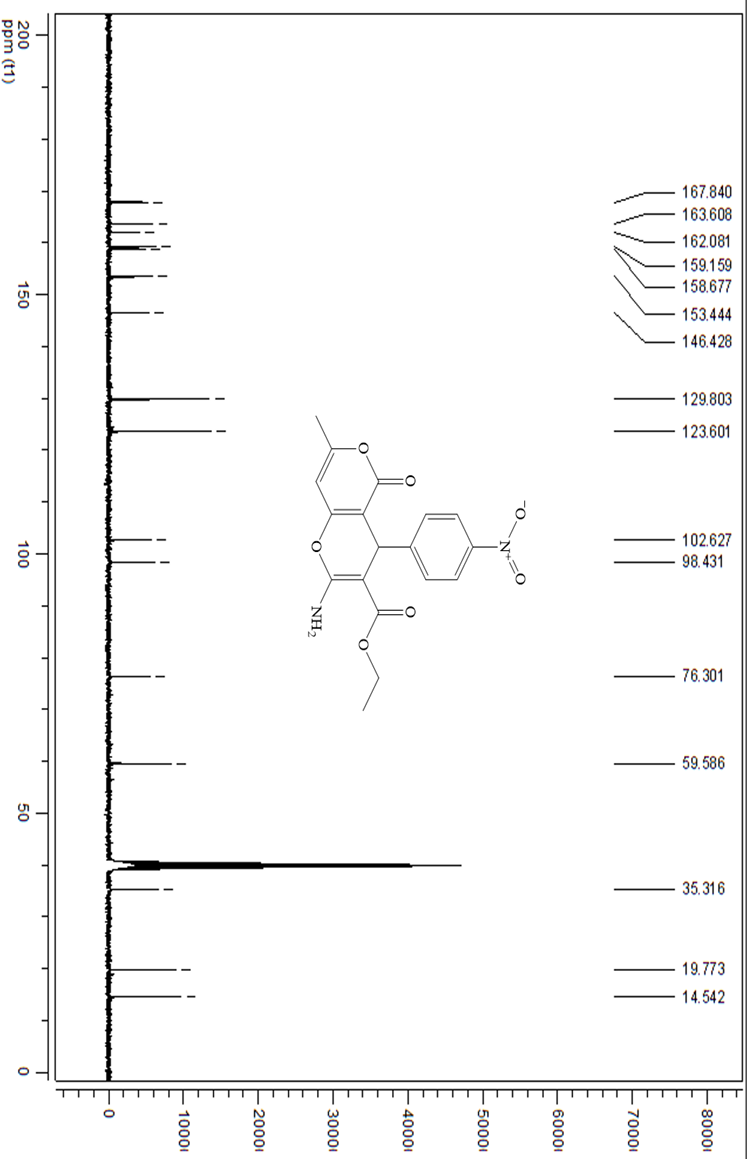


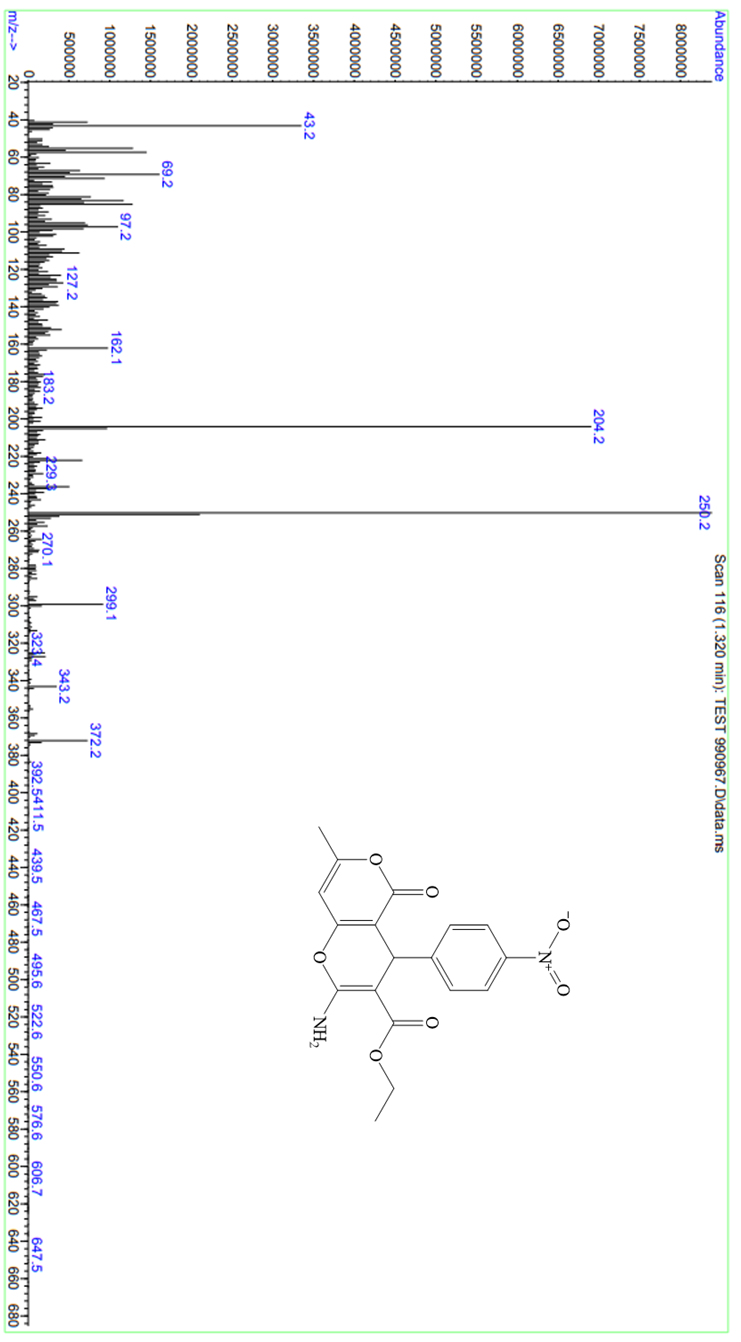


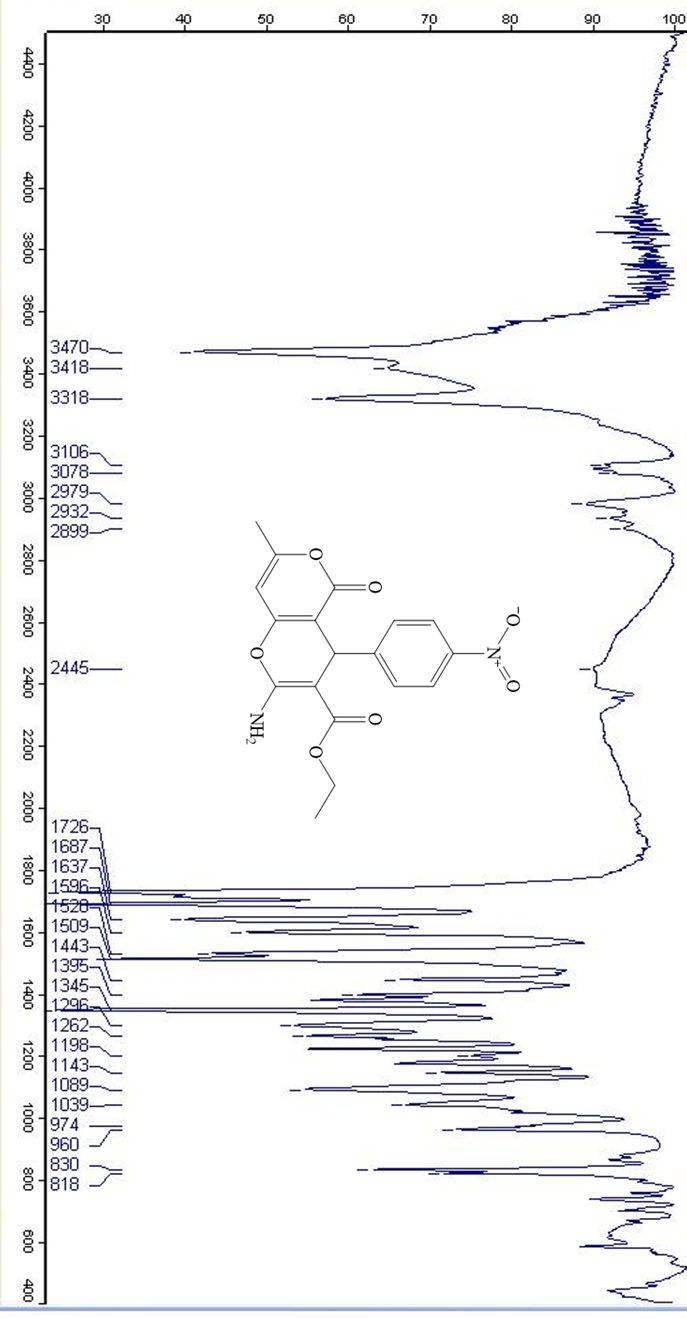


Ethyl 2-amino-7-methyl-5-oxo-4-(4-(trifluoromethyl)phenyl)-4,5-dihydropyrano [4,3-b]pyran-3-carboxylate(4h)


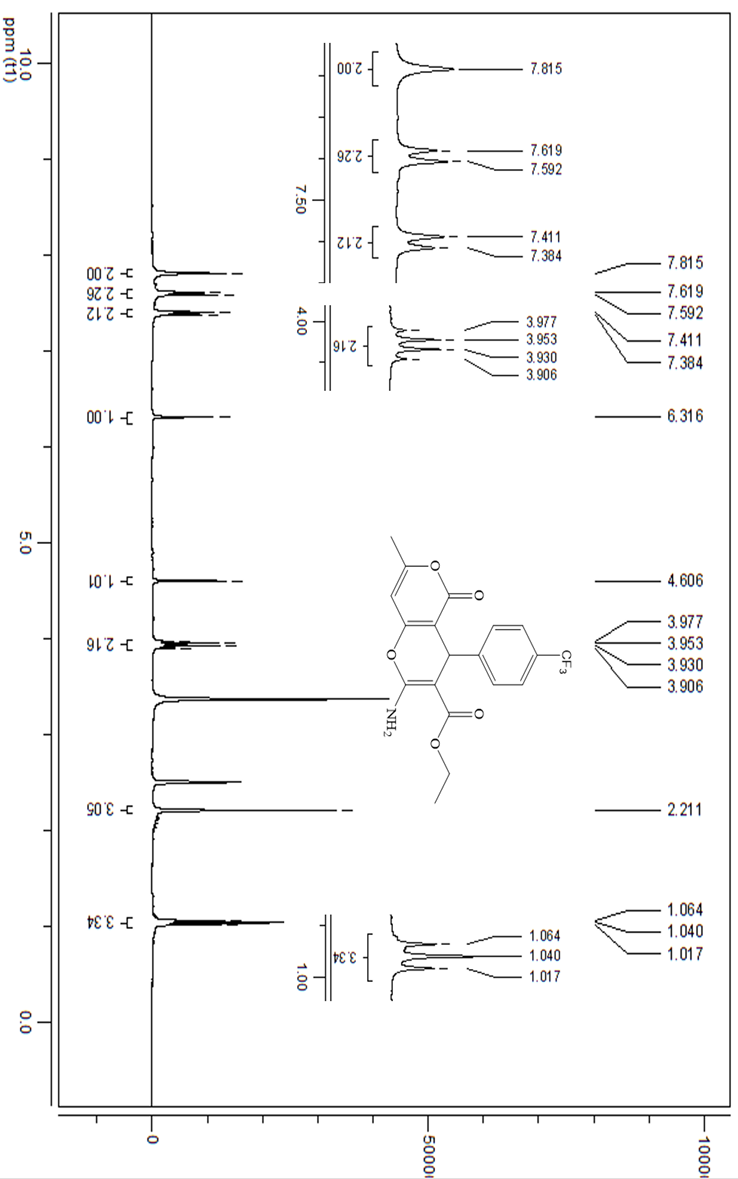


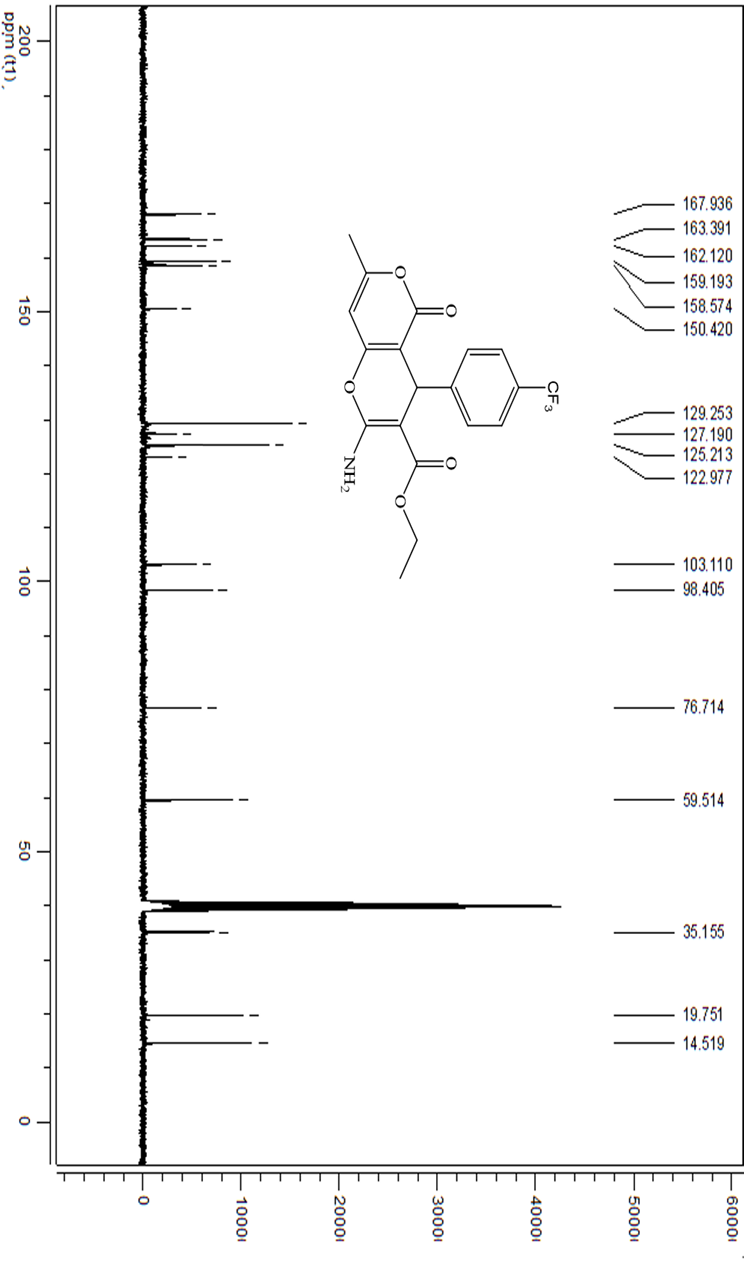


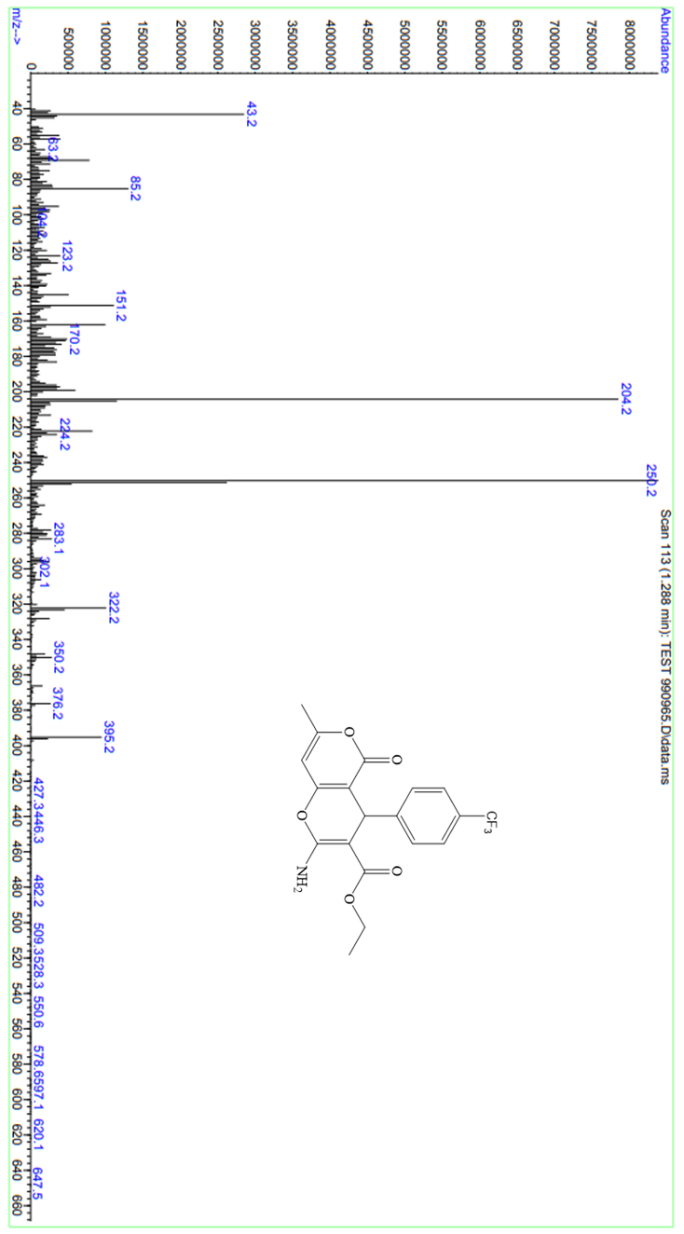


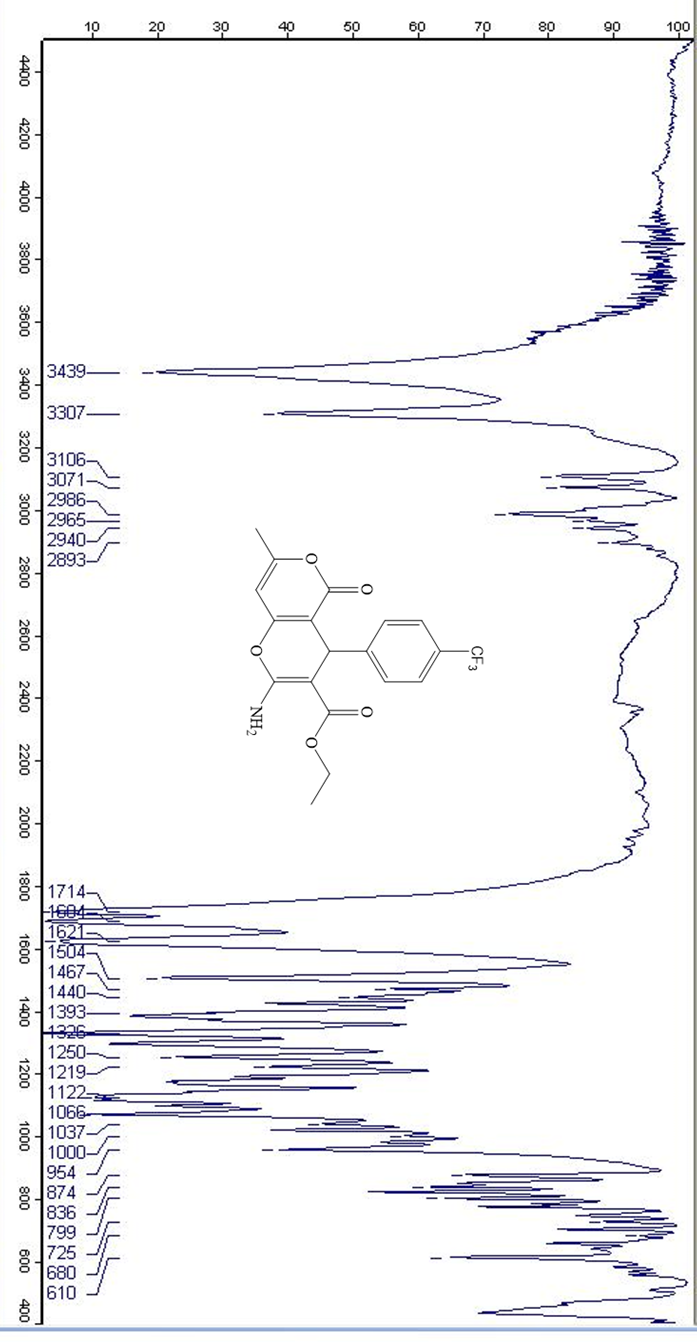


Ethyl 2-amino-4-(4-chlorophenyl)-7-methyl-5-oxo-4,5-dihydropyrano[4,3-b] pyran-3-carboxylate(4i)


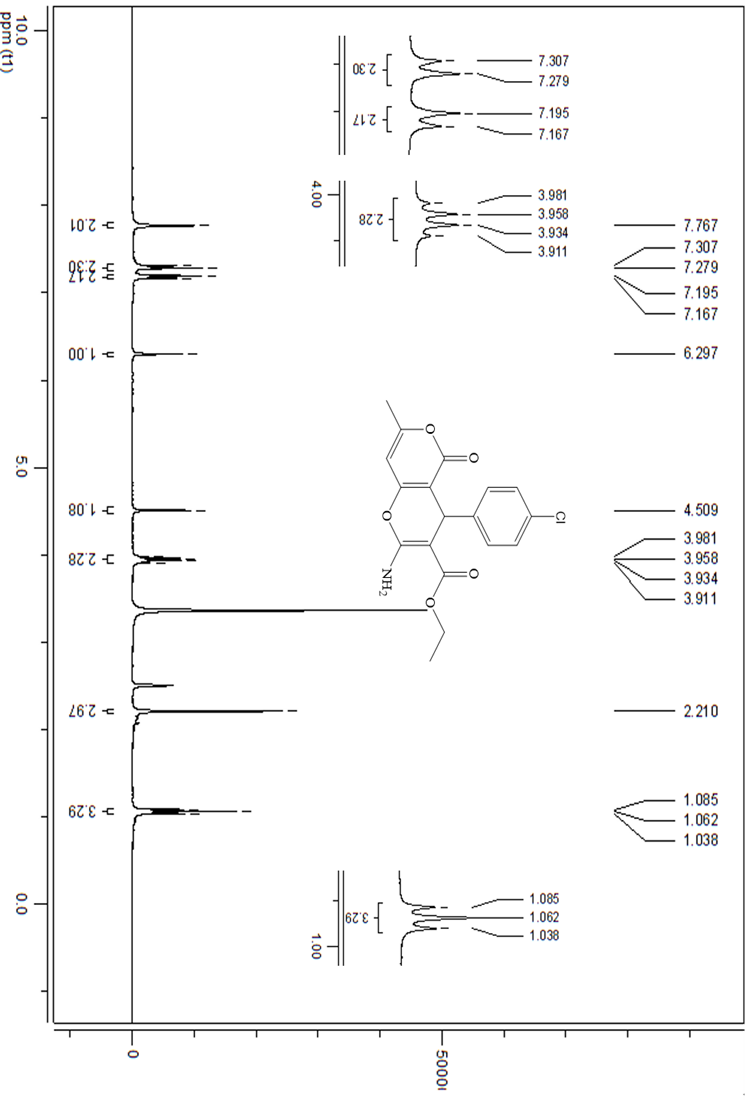


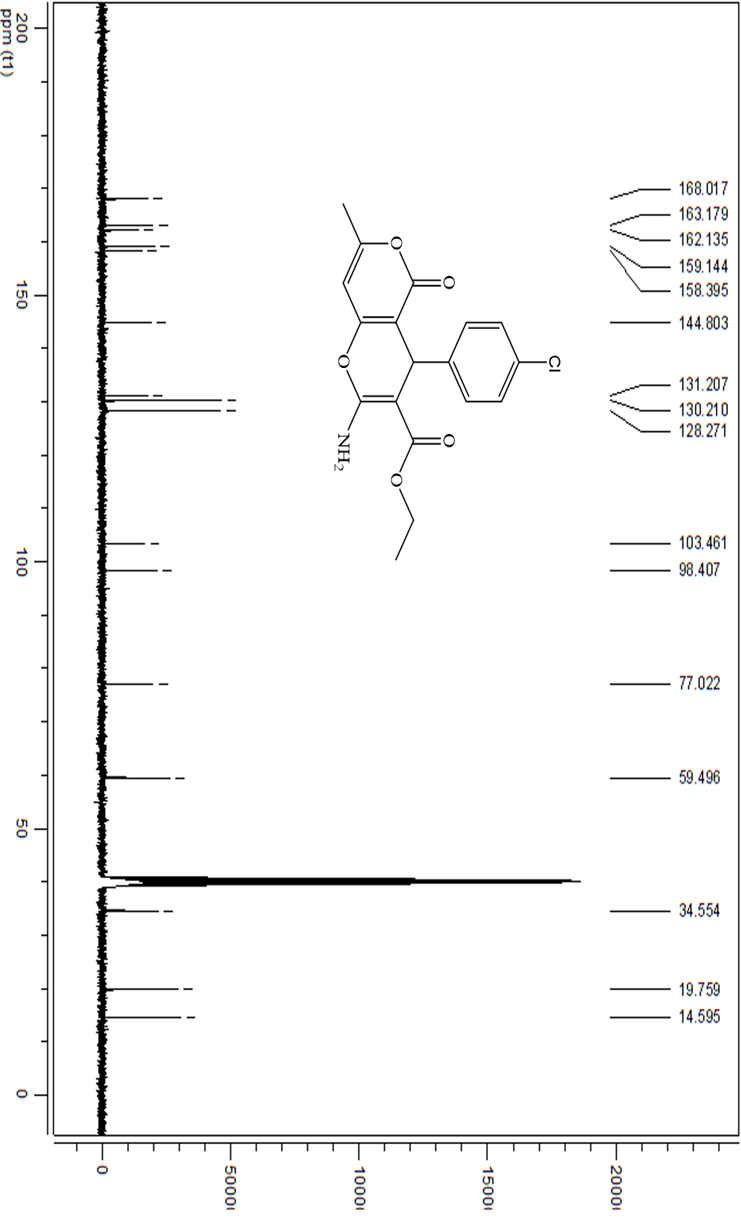


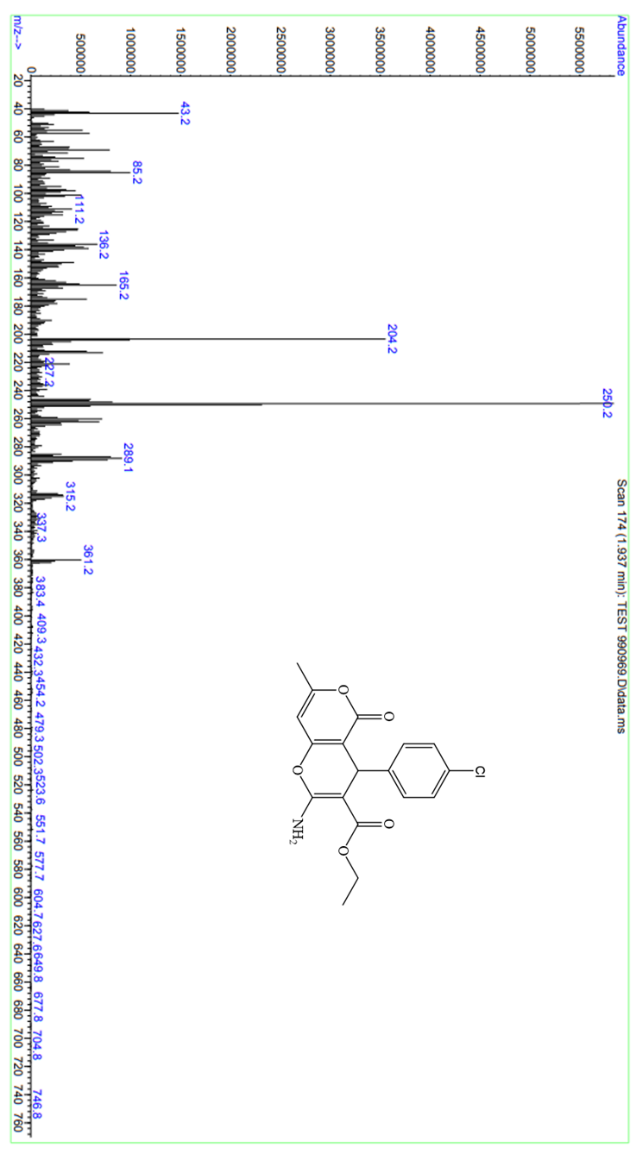


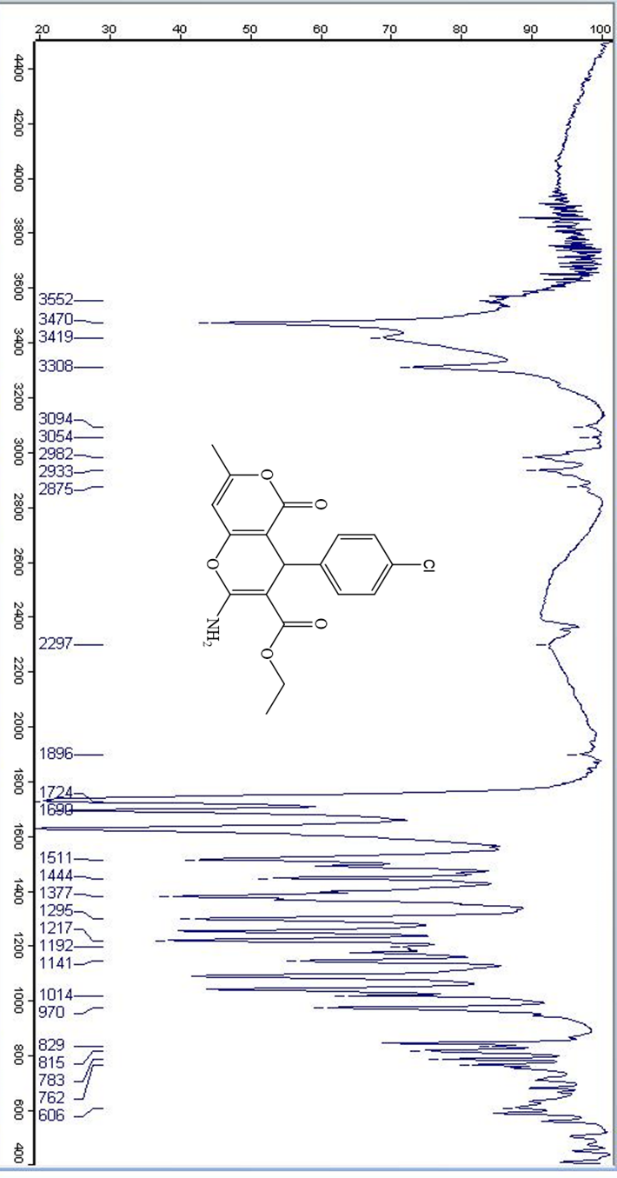


Ethyl 2-amino-7-methyl-5-oxo-4-(3,4,5-trimethoxyphenyl)-4,5-dihydropyrano [4,3-b]pyran-3-carboxylate(4j)


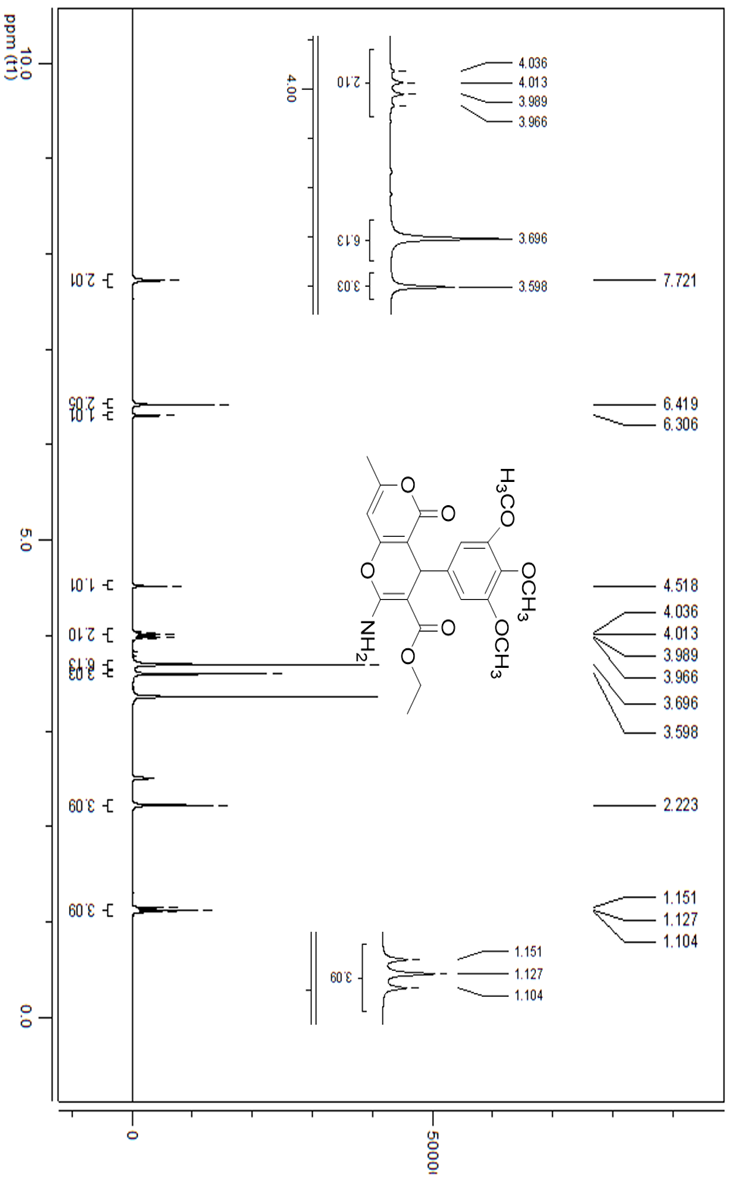


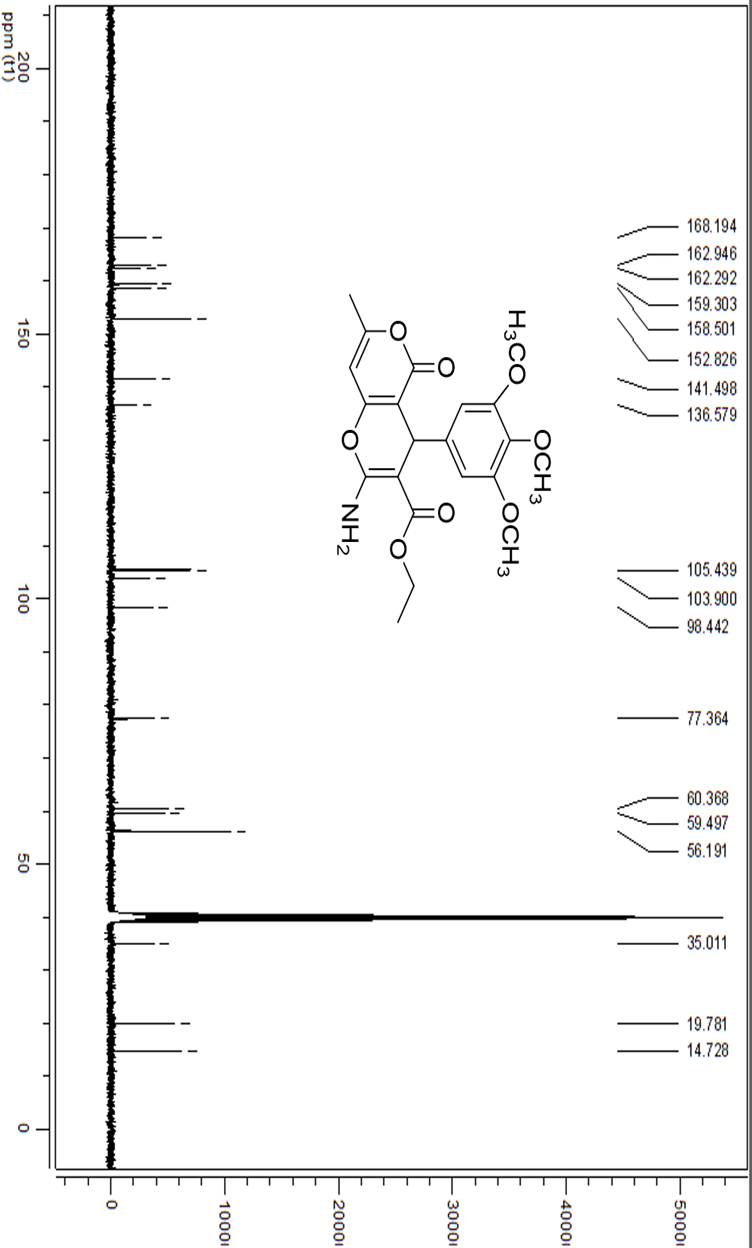


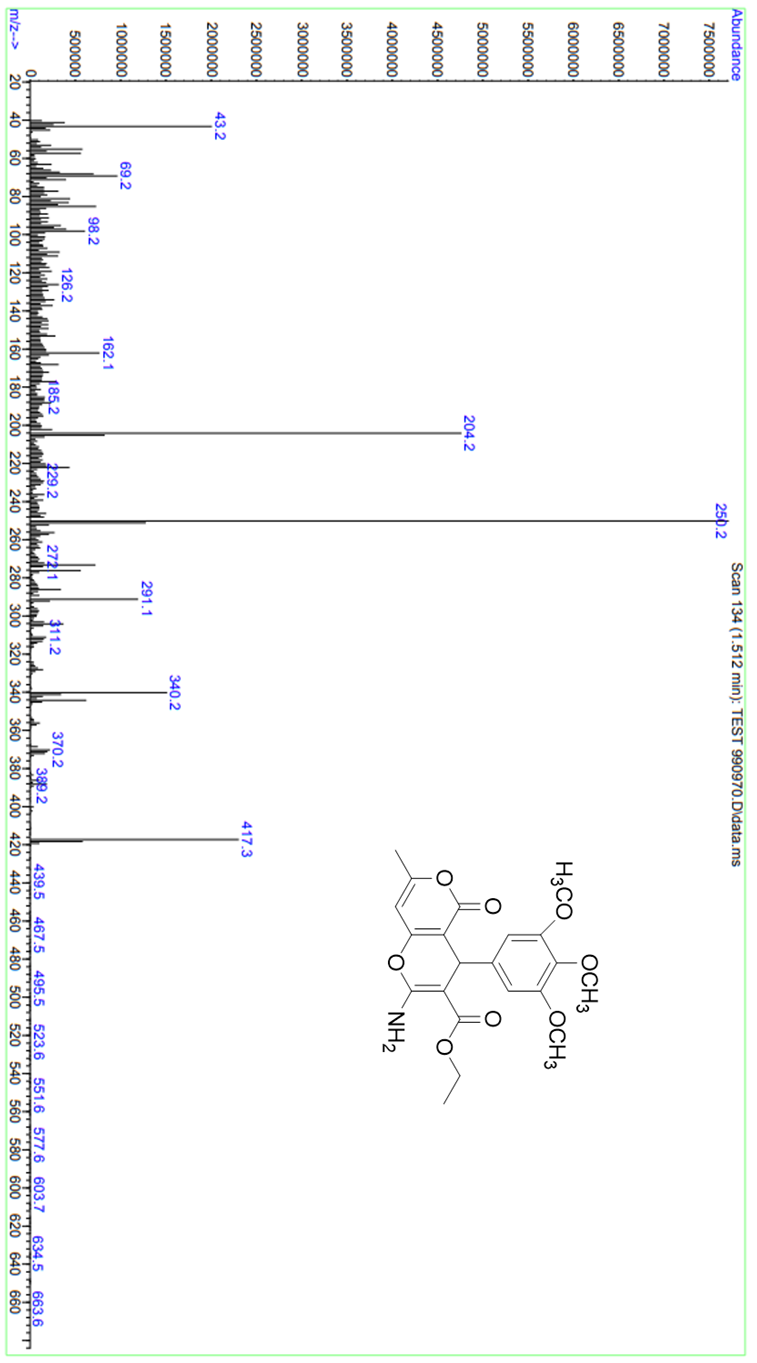


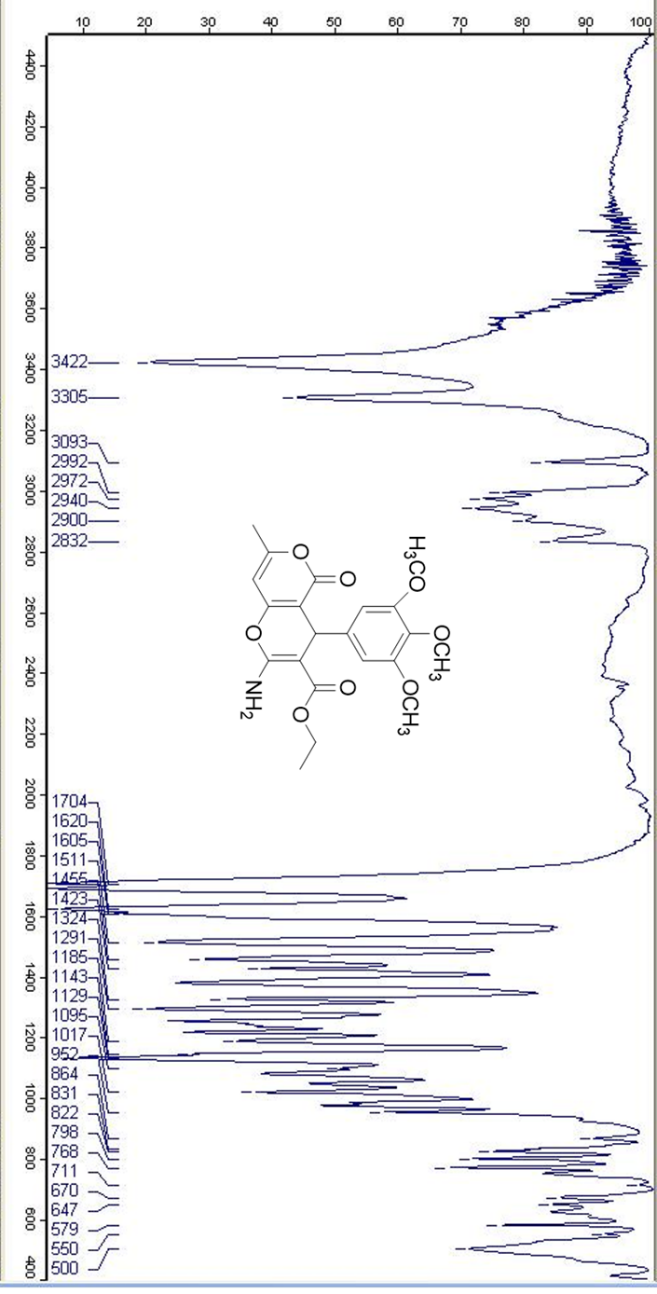

Supplement: Multimedia component 1 [file mmc1.docx]
